# Effect of sub-inhibitory tigecycline (½-MIC) on AcrAB-TolC and *mar/ram/sox* regulatory genes in *Enterobacter cloacae* complex isolates

**DOI:** 10.3389/fcimb.2026.1745642

**Published:** 2026-02-25

**Authors:** Lukasz Korczak, Piotr Majewski, Pawel Sacha, Dominika Chludzinska, Elzbieta Tryniszewska

**Affiliations:** Department of Microbiological Diagnostics and Infectious Immunology, Medical University of Bialystok, Bialystok, Poland

**Keywords:** acrAB-TolC, marA, marB, ramA, rob, soxS

## Abstract

The global rise of antimicrobial resistance (AMR) in *Enterobacterales*, including the *Enterobacter cloacae* complex, is narrowing treatment options. Tigecycline, a last-resort antibiotic for the treatment of multidrug-resistant (MDR) Gram-negative pathogens, is increasingly compromised by emerging resistance mechanisms, notably efflux pump overexpression and regulatory network adaptation. In this study, sixty clinical isolates of *Enterobacter cloacae* (thirty-eight tigecycline-resistant [TGC-R], twenty-two tigecycline-susceptible [TGC-S]) were analyzed to investigate gene expression changes in efflux pumps and regulatory genes under tigecycline pressure (1/2 minimum inhibitory concentration [MIC]) and standard conditions. Tigecycline exposure markedly increased *tolC* and *acrA* together with the regulators *marA* and *ramA*, while *acrB* increased only modestly. This indicates a strong regulatory component to the tigecycline response. In contrast, TGC-S isolates exhibited significant induction of *marA, marB* without corresponding activation of efflux pumps. Δlog_2_FC analysis highlighted distinct transcriptional shifts between exposed and unexposed groups, with resistant strains displaying greater divergence. Heatmaps and boxplot visualizations, supported by Wilcoxon test statistics, underscored the regulatory responses associated with tigecycline pressure. These findings indicate that, alongside AcrAB-TolC upregulation, stress-responsive regulators (*marA*, *ramA*) are strongly induced by sub-inhibitory tigecycline, underscoring the multifactorial regulation of tigecycline response in the *E*. *cloacae* complex.

## Introduction

The *Enterobacter cloacae* complex (ECC) is an important cause of hospital-acquired infections, particularly in intensive care units (ICU), elderly, and immunocompromised patients, and belongs to the ESKAPE group (De Oliveira et al., 2020). *E*. *cloacae* is one of the most frequently isolated species of the *Enterobacter* genus associated with clinical infections (Qureshi et al., 2011; Bazaid et al., 2022). Gram-negative bacteria, including ECC, acquire resistance through (i) activation of the efflux pumps and their regulators, (ii) enzymatic drug inactivation such as *tet*(X)-mediated tigecycline degradation, and (iii) heteroresistance (Zhong et al., 2014; Ruppé et al., 2015; Roch et al., 2023). The accumulation of such resistance determinants facilitates the emergence of MDR strains, with an increasing number of cases reported each year, constituting a major public health threat. The overexpression of efflux pumps, often modulated by global or local regulatory systems are considered key contributors to the emergence and persistence of MDR phenotype in *Enterobacter* and other *Enterobacterales* species (Davin-Regli and Pagès, 2015; Li et al., 2019).

As resistance to carbapenems continues to grow, tigecycline and colistin have emerged as critical agents of last-line therapy against MDR pathogens. Tigecycline (a 9-t-butyl glycol amide derivative of minocycline) is considered a last-resort agent for the treatment of MDR strains, particularly due to nephrotoxic and ototoxic side effects associated with colistin use (Yaghoubi et al., 2022). Its bacteriostatic activity involves inhibition of bacterial protein translation via interaction with the 30S bacterial ribosome subunit. Tigecycline possesses a wide-ranging antimicrobial spectrum of action including a variety of clinically significant species, such as: Gram-positive pathogens (*Staphylococcus aureus*, methicillin-resistant *S*. *aureus* [MRSA], *Streptococcus pneumoniae*, *Enterococcus* species, vancomycin-resistant strains [VRE]), Gram-negative microorganisms (*Enterobacterales*, extended-spectrum-beta-lactamase [ESBL] producers, and carbapenem-resistant [CRE] strains). Moreover it retains efficacy against anaerobes and atypical microorganisms (Yaghoubi et al., 2022). Natural resistance to tigecycline occurs in some bacterial species, such as *Morganella* species, *Proteus* species, *Providencia* species, and *Pseudomonas aeruginosa* (Park et al., 2015).

On the cusp of the post-antibiotic era, the incidence of bacterial resistance is increasing at an alarming rate, posing a significant threat to global public health and undermining the effectiveness of current antimicrobial therapies (Kwon and Powderly, 2021; Murray et al., 2022). Tigecycline resistance is also increasing, with reports of tigecycline-resistant bacterial strains identified even prior to its clinical introduction in China. Notably, several studies have documented TGC-R isolates of *K*. *pneumoniae* before tigecycline was officially launched in mainland China in 2011, suggesting that resistance mechanisms may be attributable to cross-resistance or regulatory elements already present in bacterial populations (Zhong et al., 2014). Resistance to tigecycline may develop via several molecular mechanisms, such as changes in OMP, mutations in regulatory gene systems, enzymatic inactivation of the antibiotic, or increased activity of the efflux pump systems (Korczak et al., 2024; Sun et al., 2014). Among the diverse molecular mechanisms contributing to tigecycline resistance, efflux pump overexpression is the most clinically significant. The AcrAB-TolC system—a member of the resistance-nodulation-division (RND) family—is the most prevalent and important efflux pump in *Enterobacterales* such as *K*. *pneumoniae* and *Escherichia coli*. Increased expression of AcrAB-TolC is often linked to mutations in regulatory genes, resulting in reduced intracellular tigecycline concentrations and elevated resistance. Although other efflux systems exist, AcrAB-TolC is considered the principal component of tigecycline resistance in clinical isolates (Sun et al., 2014).

Regulatory systems play a crucial role in the development of tigecycline resistance by controlling the expression of multidrug efflux pumps and other resistance mechanisms. Among these, the transcriptional regulators RamA, MarA, MarB, Rob, and SoxS play particularly important roles. RamA and MarA act as a key activator of efflux systems such as AcrAB-TolC, thereby reducing intracellular antibiotic concentrations and promoting survival under stress caused by antimicrobial agents (Goodarzi et al., 2021; Chirabhundhu et al., 2024). MarR functions as a repressor of the *marRAB* operon, and its inactivation can result in overexpression of MarA and increased resistance (Chirabhundhu et al., 2024). However MarB is less characterized; it is thought to modulate the activity of the *mar* operon (Wang and Li, 2024). Another transcriptional regulator associated with induction of multidrug resistance through efflux pump activation is Rob (Goodarzi et al., 2021). SoxS is induced in response to oxidative stress and can enhance efflux-mediated resistance. Exposure to sub-inhibitory concentrations of tigecycline may alter the expression of these regulators, potentially facilitating the emergence of resistant phenotypes. Sub-inhibitory tigecycline concentrations can exert selective pressure that favors mutations in regulatory genes. Such mutations may lead to constitutive expression of AcrAB-TolC in *Enterobacterales* (and, in *A*. *baumannii*, AdeABC) even after antibiotic is removed (Navidifar et al., 2019; Prieto Martin Gil et al., 2021). This adaptive mechanism is further associated with an increased prevalence driven by tigecycline, which facilitates mutations in ribosomal targets (such as *rpsJ*) and regulators associated with efflux systems, contributing to a feedback loop that reinforces resistant phenotypes (Navidifar et al., 2019; Prieto Martin Gil et al., 2021). In this context, investigating the effects of low concentrations of tigecycline on these regulatory pathways is crucial for understanding the initial stages of how resistance develops.

The aim of the study was to evaluate how ½-MIC tigecycline affects expression of efflux-associated regulators (*marA*, *marB*, *ramA*, *soxS*, *rob*) and AcrAB-TolC components in clinical ECC isolates, comparing tigecycline-resistant (TGC-R) and tigecycline-susceptible (TGC-S) groups.

## Materials and methods

### Study design and bacterial isolates

In this study, all sixty non-duplicate clinical ECC isolates were collected at a single tertiary hospital in Bialystok, Poland, between 2012 and 2023. Strain origins and clinical sources are summarized in **Table 1**. All strains were cryopreserved at -80°C in Tryptone Soya Broth (TSB; Oxoid Ltd., Basingstoke, UK) supplemented with 30% glycerol for long-term storage.

**Table 1:** The origin of strains used in the study and types of clinical material from which the strains originated.

Hospital ward	Number of isolates	% of isolates
Intensive care unit	36	60,00%
Hematology	11	18,33%
Urology	5	8,33%
Cardiology	4	6,66%
Surgery	2	3,33%
Neurology	2	3,33%
Type of material		
Respiratory tract	32	53,33%
Urine	14	23,33%
Wounds/Pus	8	13,33%
Blood	3	5,00%
Catheter	2	3,33%
Gastrointestinal tract	1	1,66%

### Detection of AmpC β-lactamases and New Delhi metallo-β-lactamases

All analyzed strains were screened for the presence of AmpC β-lactamases and New Delhi metallo-β-lactamases (NDM). AmpC production was assessed using cloxacillin-supplemented agar, with differences in inhibition zones between cefotaxime and imipenem discs serving as the evaluation criterion. AmpC production reflects clinical isolate characteristics but was not analyzed as a confounding variable, as our focus was tigecycline-specific efflux regulation. Detection of NDM strains was performed using the O.K.N.V.I. RESIST-5 *in vitro* rapid diagnostic test kit. This kit, designed for bacterial culture analysis, was purchased from Coris BioConcept (Gembloux, Belgium).

### Identification and microdilution method

Initial strains identification and MIC profiling were performed using the VITEK 2 ^®^ system (bioMérieux, Marcy-l’Étoile, France). For culture, isolates were streaked onto MacConkey agar (Oxoid Ltd., Basingstoke, UK), a selective medium for Gram-negative microorganisms, and incubated at 37°C under aerobic conditions. The MIC of tigecycline was determined by the broth microdilution method according to the European Committee on Antimicrobial Susceptibility Testing (EUCAST) guidelines (MIC determination of non-fastidious and fastidious organisms; Kelley et al., 2022). Fresh Mueller-Hinton broth (MHB) was prepared on the day of use according to the EUCAST recommendations. Tigecycline (Merck, Darmstadt, Germany) was dissolved and serially diluted in MHB to obtain a concentration range of 0,0625-64 µg/ml, allowing the detection of resistant and susceptible phenotypes (Stuart et al., 2010). Bacterial suspensions were adjusted to a 0,5 McFarland scale and diluted to obtain a final inoculum of 5x10^5^ CFU/ml (CFU, colony forming units). Each well of microdilution plate, except the negative control wells, was inoculated with bacterial suspensions. Microdilution plates were incubated under aerobic conditions at 37°C for 16 to 20 hours. Throughout the entire cultivation period, the bacterial strains in the tigecycline-exposed group were maintained under sub-inhibitory concentrations of tigecycline. Each isolate was exposed to 0,5 x its own tigecycline MIC, as determined above. The MIC is defined as the lowest concentration of tigecycline, that completely inhibited visible bacterial growth.

### Bacterial culture in standard conditions

To assess the antibiotic effects, a group of *E. cloacae* strains was prepared under standard growth conditions without antibiotic pressure. Bacterial cultures were initiated by inoculating single colonies of each strain from MacConkey agar plates into sterile MHB in the sterile test tubes. Inoculated tubes were incubated under the same conditions as microdilution plates (37°C, 16–20 hours, under aerobic conditions) to allow for overnight bacterial growth. The overnight cultures served as controls and were used for subsequent experiments.

### Bacterial RNA isolation and purification

Total RNA was extracted from both control (standard conditions) and tigecycline-exposed *E. cloacae* populations using the Total RNA Plus kit (A&A Biotechnology, Gdansk, Poland), following the manufacturer’s protocol for bacterial strains. For the tigecycline-exposed group, bacterial material was collected from cultures grown in the presence of tigecycline at a concentration corresponding to half of minimal inhibitory concentration (1/2 MIC). Following isolation, RNA was further purified and condensed using the Total RNA Plus Concentrator kit (A&A Biotechnology, Gdansk, Poland) to remove contamination. RNA concentration and purity was assessed using a NanoDrop 2000/2000c spectrophotometer (Thermo Fisher Scientific, Waltham, MA, USA).

### Reverse transcription and quantitative PCR analysis

Prior to cDNA synthesis, RNA samples were normalized to a concentration of 1000 ng to ensure equivalent input for downstream analysis. Reverse transcription was performed using the TranScriba Kit (A&A Biotechnology, Gdansk, Poland) to obtain a high-quality cDNA for further analysis. First-strand cDNA synthesis was carried out using 1000 ng of total RNA as template in a reaction mixture, according to the manufacturer’s protocol. Reactions were incubated at 25°C for 5 min, followed by cDNA synthesis at 42°C for 60 min, and enzyme inactivation at 85°C for 5 min.

Real-time quantitative PCR (RT-qPCR) reactions were performed using a SYBR Green-based master mix (A&A Biotechnology, Gdansk, Poland) with the analysis of the dissociation curve on an Agilent Mx3005P thermocycler (Agilent Technologies, Waldbronn, Germany). Thermal cycles conditions included the following: initial denaturation (95°C for 5 min), forty cycles at 95°C for 15 s, annealing at 55°C for 30 s, and elongation at 72°C for 30–40 s, followed by final elongation at 72°C for 5 min. Primers for target and reference genes were designed using NCBI Primer-BLAST and validated to ensure specificity and efficiency. The complete list of oligonucleotides is provided in **Supplementary Table S1** in **Supplementary Materials** (Majewski et al., 2020). Analyzed genes included the following: primary AcrAB-TolC regulatory genes (*ramA*, *marA*, *marB*, *rob*, *soxS*), and efflux pump genes (*acrA*, *acrB*, *tolC*). To ensure reliable normalization of gene expressions, the *rpoB* gene encoding the β subunit of bacterial RNA polymerase was used as a reference gene. This gene is recognized for stable expression across bacterial species and experimental conditions; *rpoB* stability was confirmed across exposed and unexposed conditions (Ct variation <1 cycle in a subset of isolates.

### Relative gene expression analysis

Expression levels of target genes in standard conditions and under tigecycline exposure were normalized to the *rpoB* gene using the Pfaffl method, which provides a more accurate quantification of qPCR data by accounting for differences in amplifications between target and reference genes (Pfaffl, 2001). The Pfaffl method also corrects for variations in the efficiency of the qPCR reaction, thus minimalizing potential bias in the results. *E. cloacae* ATCC 700323 (Oxoid Culti-Loops, Basingstoke, UK) was used as a molecular reference. Baseline expression in the reference strain remained low, so even modest induction under tigecycline produced large log_2_-transformed fold changes relative to that baseline. Relative expression was computed using the efficiency-corrected Pfaffl model with primer-specific amplification efficiencies, ensuring that the observed amplitudes are not artifacts of unequal PCR efficiency between targets and references.

Fold changes (FCs) in gene expressions were log2-transformed to normalize data distribution and facilitate comparative analysis. For interpretation of expression dynamics, three distinct categories were established as follows: downregulation: log_2_FC ≤ −1.0; unchanged: −1.0 < log_2_FC < 1.0; upregulation: log_2_FC ≥ 1.0. This classification aligns with established practices for identifying meaningful transcriptional shifts while accounting for technical variability inherent in qPCR assays.

### Statistical analysis

Descriptive statistical measures, including median, mean, minimum, maximum and standard deviation were calculated for gene expression in both populations. All visuals including boxplots and heatmaps were prepared using GraphPad Prism 10.5.0 (GraphPad Software Inc., San Diego, CA, USA). Statistical comparison of gene expression between standard conditions and after tigecycline exposure, within each phenotype (resistant and susceptible strains), were performed using the Wilcoxon matched-pairs signed rank test. No correction for multiple comparisons was applied. Differences in the response to tigecycline between resistant and susceptible isolates were assessed by calculation of Δlog_2_FC in each subpopulation in both conditions (log_2_FC after tigecycline exposure minus log_2_FC under standard conditions). STATA 17 (StataCorp, 2021) was used to assess this data (StataCorp LP, 2021).

## Results and discussion

**Figure 1:**
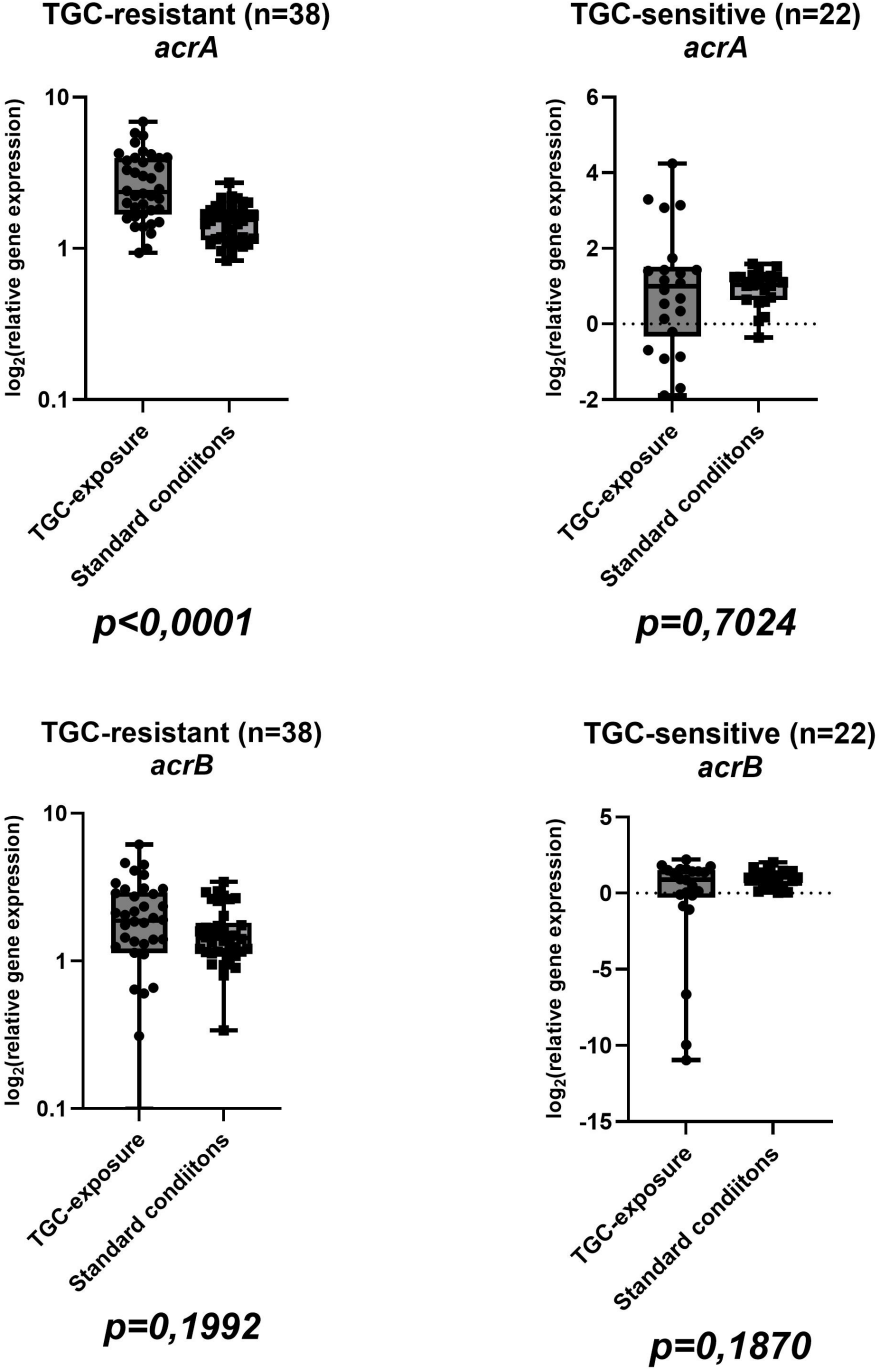
Boxplots illustrating the distribution of *acrA* and *acrB* gene expression in TGC-R (n=38) and TGC-S (n=22) isolates under tigecycline exposure/standard conditions. Each point represents one clinical isolate. *P*-values from the Wilcoxon test are indicated below each comparison (same isolate ± tigecycline).

**Figure 2:**
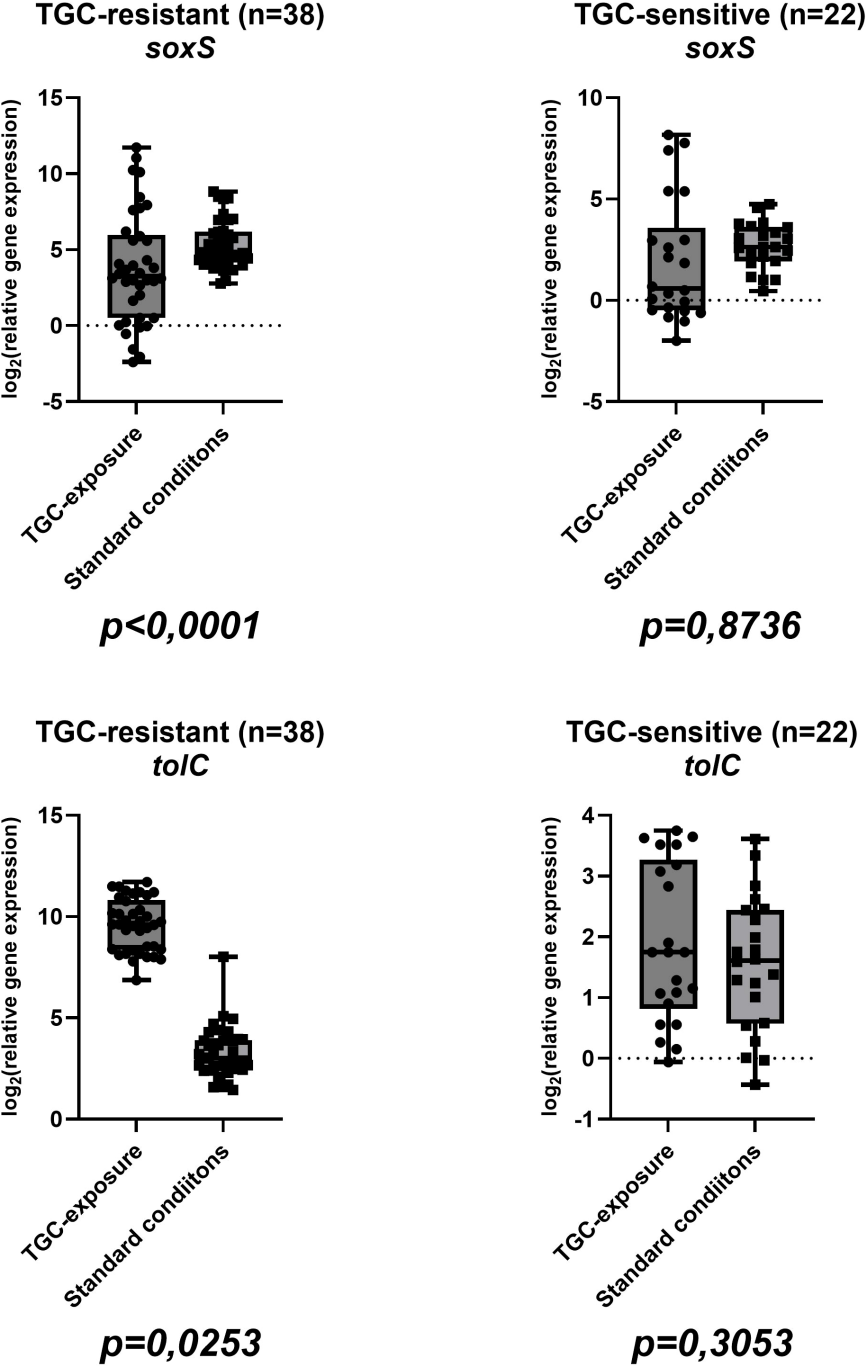
Boxplots illustrating the distribution of the *marA* and *marB* genes expression in TGC-R (n=38) and TGC-S (n=22) isolates under tigecycline exposure/standard conditions. Each point represents one clinical isolate. *P*-values from the Wilcoxon test are indicated below each comparison (same isolate ± tigecycline).

**Figure 3:**
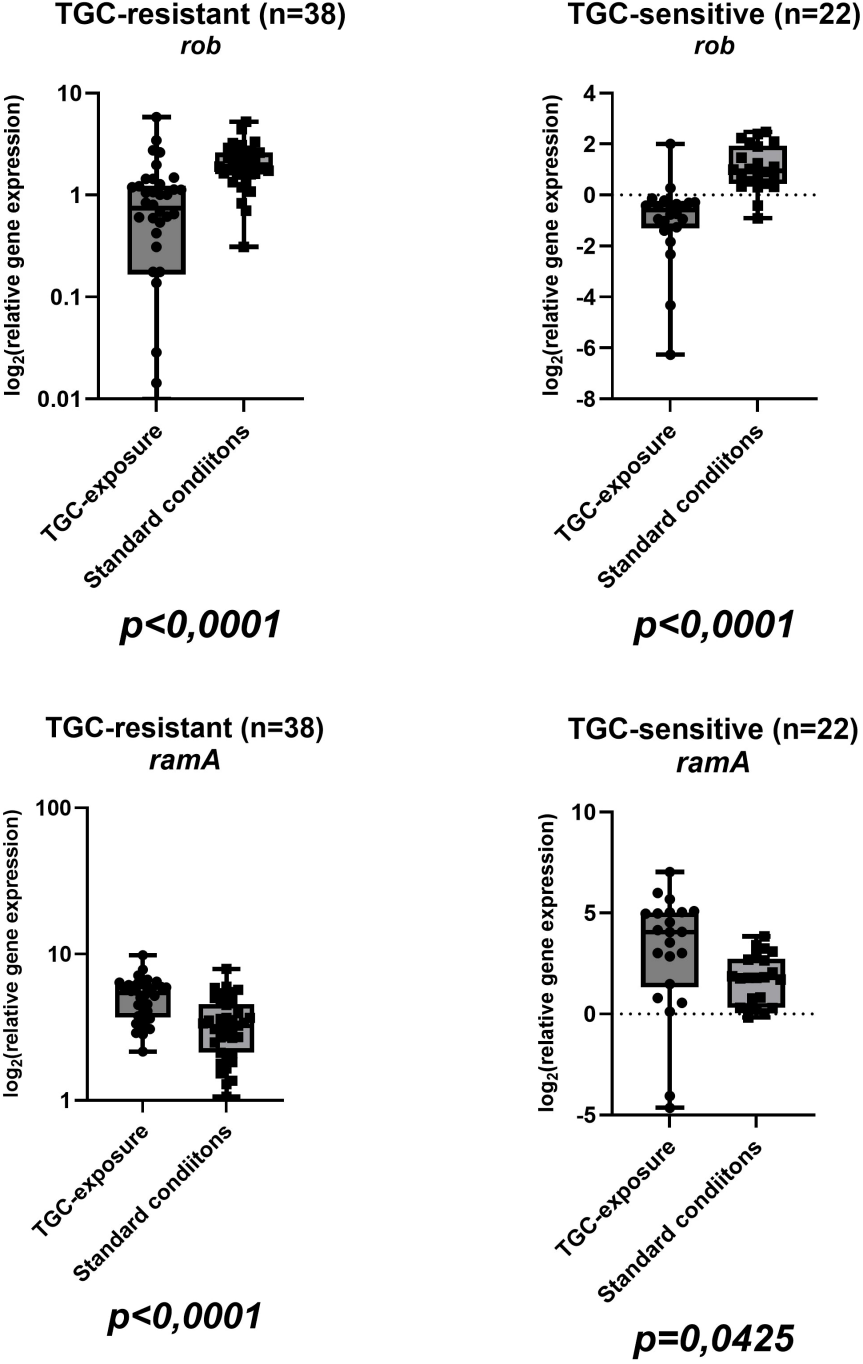
Boxplots illustrating the distribution of regulatory genes *ramA* and *rob* expressions in TGC-R (n=38) and TGC-S (n=22) isolates under tigecycline exposure/standard conditions. Each point represents one clinical isolate. *P*-values from the Wilcoxon test are indicated below each comparison (same isolate ± tigecycline).

**Figure 4:**
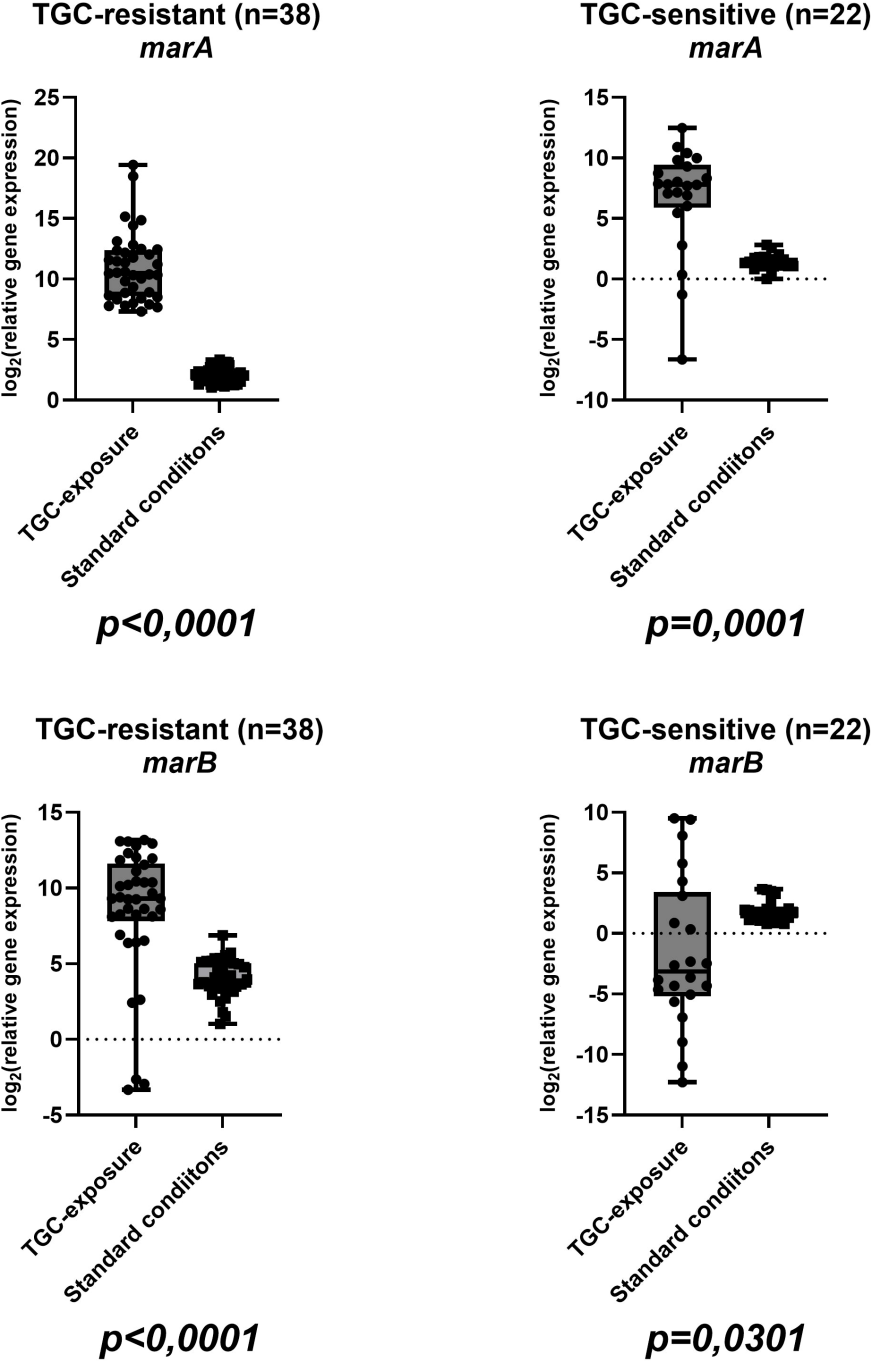
Boxplots showing log_2_-relative expression levels of *soxS* and *tolC* genes in TGC-R (n=38) and TGC-S (n=22) isolates under tigecycline exposure/standard conditions. Each point represents one clinical isolate. Wilcoxon test *p*-values are provided for each comparison (same isolate ± tigecycline).

Sixty isolates were included in this study, comprising thirty-eight (63,33%) TGC-R and twenty-two (36,67%) TGC-S. Among tigecycline-resistant isolates, twenty-two of thirty-eight (57.9%) harbored AmpC β-lactamase, whereas four of eighteen (22.2%) tigecycline-susceptible isolates were AmpC-positive; NDM was not detected in any isolate. The mean tigecycline MIC values were 12 ± 7,8 µg/ml (range: 4-32 µg/ml) for resistant isolates and 0,5 ± 0,3 µg/ml (0,25-1 µg/ml) for susceptible strains (**Table 2**). Complete gene expression data for all isolates in both conditions are visualized in boxplots in **Figures 1**, **2**, **3**, **4** and summarized in **Supplementary Tables S2–S7** in **Supplementary Materials**. In tigecycline-resistant isolates exposed to ½-MIC tigecycline, the highest mean log_2_FC values were observed for *marA* (10,97) and *marB* (5,11), whereas *rob* (0,77) and *acrB* (1,92) showed only modest induction. In contrast TGC-S isolates exhibited the highest expression in *marA* (6,68) and *ramA* (3,06), with the lowest means in *rob* (-1,02) and *acrB* (0,51). Under standard conditions, the highest mean values in TGC-R isolates were observed in regulatory gene *soxS* (5,31), while the lowest was observed in efflux pump genes *acrA* (1,52), and *acrB* (1,61). In the TGC-S group *soxS* showed moderate induction (2,68), with the minimal changes in genes coding efflux pump genes (*acrA*: 0,94; *acrB*: 0,95). Complete basic statistical values are presented in **Table 3** and visualized in **Figures 1**, **2**, **3**, **4** (boxplots showing log2FC distributions with Wilcoxon test *p*-values for all genes tested).

**Table 2:** Tigecycline MIC values of TGC-R and TGC-S isolates (median ± SD, μg/mL).

Subpopulation	MIC values of tigecycline (median ± SD)
TGC-R (n=38)	8 ± 7,8 μg/mL
TGC-S (n=22)	1 ± 0,3 μg/mL

**Table 3:** Fold Change (FC) gene expression values across experimental and standard conditions (median ± SD).

Gene	FC gene expression (median ± SD)			
	TGC-R		TGC-S	
	TGC-exposed	Standard	TGC-exposed	Standard
*acrA*	5 ± 21	3 ± 1	2 ± 4	2 ± 1
*acrB*	4 ± 12	3 ± 2	2 ± 1	2 ± 1
*ramA*	43 ± 145	9 ± 40	16 ± 29	4 ± 3
*rob*	2 ± 9	4 ± 6	1 ± 1	2 ± 1
*marA*	1446 ± 127800	4 ± 2	222 ± 1222	3 ± 1
*marB*	634 ± 2804	15 ± 21	1 ± 210	3 ± 3
*tolC*	328 ± 888	8 ± 41	65 ± 4	3 ± 3
*soxS*	10 ± 672	29 ± 109	2 ± 80	6 ± 6

In the tigecycline-exposed group, TGC-R strains exhibited widespread upregulation of key genes, including efflux pump components (*acrA*, *acrB*, *tolC*), and regulatory genes (*ramA*, *marA*, *marB*, *soxS*). Notably, *rob* – a gene associated with bacterial oxidative stress response – remains unchanged under these conditions unlike other regulators (*e.g. ramA*). The *rob* gene encodes a transcriptional regulator belonging to the XylS/AraC family, known to activate efflux pump systems and enhance multidrug resistance and response to environmental signals, such as oxidative stress (Pérez et al., 2012). *SoxS* is a part of the *soxSR* redox-responsive regulator, which has been well described in *E*. *coli* and other *Enterobacterales*. Products of the induced *soxSR* are responsible for avoiding the eventual oxidative damage or repairing it using various mechanisms, including scavenging of oxidants, DNA repair, reconstitution of NADPH pool, excretion of toxicants (by efflux pumps) or reduced permeability. In the tigecycline-exposed TGC-R group, the *soxS* gene was upregulated (log_2_FC=3,78; **Figure 4**), aligning with prior observations in *Enterobacterales*, where a five-fold increase in *soxS* expression was associated with activation of AcrAB-TolC efflux pump and *mdtG* multidrug transporter, a mechanism strongly correlated with resistance to fluoroquinolones, tetracyclines and chloramphenicol (Fàbrega et al., 2009). A study of *E. cloacae* clinical isolates (strain EcDC64) demonstrated a significant correlation between AcrAB-TolC efflux pump overexpression and upregulation of the *soxS* (Pérez et al., 2012). Furthermore, these strains exhibited eight- to sixteen-fold higher MIC values for ciprofloxacin and tetracycline compared to *soxS*-null mutants, and downregulated *soxS* expression markedly reduced efflux activity. Together, these findings and our data underscore the role of *soxS* in mediating efflux-driven resistance in *Enterobacterales*. The coordinated *soxS-acrAB* axis appears to represent an evolutionary conserved mechanism in *E. cloacae* to limit intracellular drug accumulation during tigecycline exposure. The apparent decrease in *rob* and *soxS* in the Δlog_2_FC analysis likely reflects higher baseline expression of these genes in TGC-R isolates under unexposed conditions, rather than true repression by tigecycline.

Beyond *soxS* our data revealed significant upregulation of the *marA and marB* in TGC-R strains exposed to tigecycline. These genes comprising the activator *marA*, and *marB*, which are a well-documented driver of multidrug resistance in *Enterobacterales*. Notably, *marA* overexpression has been linked to efflux pump activation, porin downregulation and cross-resistance to β-lactams, tetracyclines, and quinolones (Chollet et al., 2002; Holden and Webber, 2020). Tigecycline-resistance in our isolates may therefore involve *marA*-mediated mechanisms alongside *ramA*, as shown in *Enterobacter* and *Salmonella* models where *marA* upregulation enhanced efflux activity and reduced drug susceptibility (Veleba and Schneiders, 2012; Sharma et al., 2017; Davin-Regli et al., 2021). The marked upregulation of the *marA* and *marB* in TGC-R-exposed strains (**Figure 2**) correlates with its established role in multidrug resistance in *Enterobacterales*. MarA, as a central transcriptional activator drives efflux pump systems like AcrAB-TolC, synergistically reducing intracellular drug concentrations (Veleba et al., 2013; Wang and Li, 2024). MarB, although less characterized, appears to modulate operon activity, and may stabilize regulatory feedback loops under antibiotic pressure (Vinué et al., 2013). This operon’s activity intersects with *ramA*, another key regulator of AcrAB-TolC, which also showed elevated expression in TGC-R exposed strains (**Figure 3**). In *E*. *cloacae*, *ramA* and *marA* synergistically amplify efflux pump activity, creating a redundant regulatory network that ensures robust resistance phenotypes (Veleba et al., 2013; Wang and Li, 2024). Such genetic redundancy allows strains resistant to tigecycline to maintain efflux-mediated resistance even if one regulator is compromised, as demonstrated in clinical isolates where *ramR* mutations and *marA* overexpression independently drive tigecycline resistance (Veleba et al., 2013). The co-upregulation of *marA* and *ramA* genes in our findings may suggest an adaptive strategy to tigecycline-induced stress (oxidative and antibiotic), highlighting the adaptability of *Enterobacterales* in clinical isolates.

The emergence of multidrug resistance in *E*. *cloacae* complex is closely associated with overexpression of efflux pump systems, particularly the AcrAB-TolC tripartite complex, which extrudes a wide range of structurally diverse compounds (Pérez et al., 2007). Recent studies have shown that the transcriptional activation of *acrAB-tolC* genes is tightly regulated by multiple global regulators, including MarA, SoxS, and RamA, whose expression can be significantly altered in response to antibiotic exposure, including sub-inhibitory concentrations (Pérez et al., 2012; Li et al., 2015). Our transcriptional profiling of the TGC-R-exposed group revealed an upregulation of efflux pump genes (mean log_2_FC: *acrA* = 2,85; *acrB* = 1,93; *tolC* = 9,57; **Figures 1** and **4**), consistent with the role of AcrAB-TolC in tigecycline resistance. Exposure to sub-inhibitory concentrations of various antibiotics has been shown to markedly affect efflux gene expression, and in *A*. *baumannii* such exposure modulated the activity of RND efflux promoters in a strain- and antibiotic-dependent manner (Ebbensgaard et al., 2020; Prieto Martin Gil et al., 2021). Similarly, antibiotics that are AcrAB-TolC substrates can upregulate transcriptional regulators: in *Salmonella choleraesuis*, selection of mutants on enrofloxacin-, ampicillin-, oxytetracycline-, and kanamycin-containing plates resulted in increased *acrB* expression (1,6-7,7-fold compared to parental isolates) and significant upregulation of *ramA* (3,2-55,9-fold) (Usui et al., 2013). Reduced tigecycline susceptibility in *E*. *cloacae* has also been linked to RamA-mediated AcrAB pump overexpression; inactivation of *acrA* or *acrB* restored tigecycline susceptibility, while genetic complementation reverted to the original resistance phenotype (Ruzin et al., 2005).

In the TGC-S group exposed to tigecycline, most regulatory genes displayed increased expression. Notably, *ramA* (**Figure 3**), and *marA* (**Figure 2**) were significantly upregulated, while *soxS* showed moderate increase (**Figure 4**). In contrast, *marB* (**Figure 2**) and *rob* (**Figure 3**) were downregulated. Meanwhile, the expression of the AcrAB-TolC efflux pump remained unchanged or slightly reduced (**Figures 2**, **4**), indicating activation of regulatory pathways, with comparatively weaker induction of structural efflux components. Studies of clinical *K*. *pneumoniae* strains have shown that in susceptible strains, the expression of *marA*, *soxS*, and *rarA* did not significantly differ from baseline, and that upregulation of these regulators and their efflux systems is primarily observed in resistant isolates, often as a consequence of mutations in regulatory genes such as ramR or acrR. Likewise, the response of efflux pump genes to sub-inhibitory tigecycline concentrations in susceptible backgrounds is variable and strain-dependent (He et al., 2015).

To more reliably assess transcriptional changes between tigecycline-exposed and unexposed groups, we quantified global expression differences using a Δlog_2_FC distance metric. In the TGC-R group (exposure *vs*. standard conditions), efflux pump components exhibited marked upregulation compared to baseline, with mean Δlog_2_FC values of 1,33 (*acrA*), 0,32 (*acrB*), and 6,31 (*tolC*). Among regulatory genes, *marA* (Δlog_2_FC = 8,97), *marB* (4,51), and *ramA* (1,70) were strongly induced, while *rob* (-1,32) and *soxS* (-1,53) exhibited downregulation. These values, summarized in bar charts in **Supplementary Figures S1–S4**, underscore the coordinated activation of efflux systems, and distinct regulatory dynamics in resistant strains upon tigecycline pressure.

To visually assess the transcriptional differences between tigecycline-exposed and baseline groups, boxplots were generated for all genes tested, illustrating their expression distributions across experimental conditions. The Wilcoxon signed-rank test, a non-parametric method robust to non-normal data was applied to assess statistical differences in median expression levels in TGC-R groups (exposed to antibiotic *vs*. baseline) and for TGC-S groups in the same conditions. In TGC-R groups exposure to tigecycline elicited a functionally stratified transcriptional response. Efflux pump components exhibited variable regulation: *acrA* (*p* < 0,0001), and *tolC* (*p* = 0,0253) were significantly upregulated, whereas *acrB* expression remained unchanged (*p* = 0,1992). Among regulatory genes, *marA* (*p* < 0,0001), *marB* (*p* < 0,0001), *ramA* (*p* < 0,0001), *rob* (*p* < 0,0001) and *soxS* (*p* < 0,0001) exhibited robust induction, underscoring their central role in coordinating resistance. These findings suggest that TGC-R strains under exposure prioritize regulatory network activation (via *mar*/*sox* systems) to sustain efflux-mediated resistance. The lack of *acrB* induction despite *acrA*/*tolC* upregulation may reflect post-transcriptional regulation or compensatory redundancy in efflux pumps.

In TGC-S groups, exposure to tigecycline did not significantly alter expression of efflux pump genes (*acrA*: *p* = 0.7024; *acrB*: *p* = 0.1870; *tolC*: *p* = 0.3053) or *soxS* (*p* = 0.8736). In contrast regulatory genes were significantly upregulated: *marA* (*p* = 0.0001), and *marB* (*p* = 0.0301); *ramA* (*p* = 0,0425) and *rob* (*p* < 0,0001). The activation of the *marA* and *marB* in TGC-S strains therefore appears to represent a stress response distinct from efflux pump – mediated resistance, potentially priming bacteria for adaptation. Unlike resistant isolates, where *mar/sox* activation drives efflux pumps, susceptible strains prioritize transcriptional regulation over efflux under tigecycline pressure. Taken together, these findings suggest that, in TGC-S strains tigecycline exposure mainly triggers regulatory adaptations rather than efflux-mediated resistance.

### Clinical implications and future perspectives

Our findings demonstrate that sub-MIC (1/2 MIC) concentrations of tigecycline induce significant upregulation of the AcrAB-TolC efflux pump and associated stress regulators in clinical *Enterobacter cloacae* complex strains, including both tigecycline-susceptible and tigecycline-resistant isolates. This mechanism contributes to low-level resistance phenotype that facilitates the transition from phenotypic tolerance to full clinical resistance, a process increasingly documented in MDR Gram-negative infections treated with last-resort antimicrobials. Clinically, sub-MIC exposure may occur in tissue niches with suboptimal drug penetration or during prolonged/suboptimal dosing regiments, potentially driving efflux-mediated treatment failure, as observed in *Enterobacter* bloodstream and pneumonia cases (Kelesidis et al., 2008).

These results have direct implications for antimicrobial stewardship – tigecycline dosing should aim to maintain concentrations above MIC to minimize selective pressure on efflux systems. Routine monitoring of MIC values in *Enterobacterales* surveillance programs is also recommended. Future studies should validate sub-MIC tigecycline effects using *in vivo* infection models and test adjunctive therapies, including efflux pump inhibitors (*e.g.* PaβN derivatives) applied to investigate their ability to restore susceptibility and limit resistance emergence. Moreover, expanding the gene panel to include additional regulators and assessing correlations across multiple regulatory systems through transcriptional network analysis would offer even deeper comprehensive insights.

Several limitations should be considered when interpreting the findings of this study. First, the analysis was conducted on a finite collection of clinical *E*. *cloacae* isolates, which may not fully capture the diversity present in clinical and environmental populations. Second, gene expression was assessed at a single time point following tigecycline exposure; thus, dynamic or transient transcriptional changes occurring at earlier or later stages may have been missed. Third, the study focused on mRNA expression levels, which do not always directly correlate with protein abundance or functional activity, especially in the context of post-transcriptional and post-translational regulation. Finally, the use of sub-inhibitory tigecycline concentrations, while relevant for modeling clinical exposure, may not reflect the full spectrum of antibiotic pressures encountered *in vivo*.

## Data Availability

The original contributions presented in the study are included in the article/**Supplementary Material**; further inquiries can be directed to the corresponding author.
