# Supplementary material for: Effect of sub-inhibitory tigecycline (½-MIC) on AcrAB-TolC and mar/ram/sox regulatory genes in Enterobacter cloacae complex isolates
Source: Front Cell Infect Microbiol. 2026 Feb 25;16:1745642. doi: 10.3389/fcimb.2026.1745642 (PMC12975997; doi:10.3389/fcimb.2026.1745642)
Supplement: Supplementary file 1 [file DataSheet1.docx]

| **Oligonucleotide** | **Sequence (5’- 3’)** | **Product** | **Application** | **Reference** |
| --- | --- | --- | --- | --- |
| **rpoB-Fq** | TCCACTCATGACGGACAACG | 170 bp | qPCR | **(23)** |
| **rpoB-Rq** | GCCATGAACCACGGTAAGGA |  | qPCR | **(23)** |
| **ramA-Fq** | GGGGAGAGCAGTATGACCATTT | 151 bp | qPCR | **(23)** |
| **ramA-Rq** | TGTATTGCATAAACAGCCGCTG |  | qPCR | **(23)** |
| **marA-Fq** | CAGACGCAATACTGACGCTA | 120 bp | qPCR | **(23)** |
| **marA-Rq** | AGGTGCCATTTGGAGTAACC |  | qPCR | **(23)** |
| **soxS-Fq*** | TTTACAGAGAATGTTCCGCACC | 124 bp | qPCR | **(23)** |
| **soxS-Rq*** | CATGGCGATATCAAAAATGGGC |  | qPCR | **(23)** |
| **rob-Fq** | CCGATTCTGTATGGTTTGAACGAA | 163 bp | qPCR | **(23)** |
| **rob-Rq** | CGTATGAGAACATCACATACTCGC |  | qPCR | **(23)** |
| **tolC-Fq** | CATTCAGGATGTCACCTATCAGAC | 147 bp | qPCR | **(23)** |
| **tolC-Rq** | GTGGTTTGATCTAACTGACGGTAA |  | qPCR | **(23)** |
| **acrA-Fq** | AAGCGGTATTATCCTGAAACGTAA | 178 bp | qPCR | **(23)** |
| **acrA-Rq** | CTTCTTGTAACGATTCAACGTCAG |  | qPCR | **(23)** |
| **acrB-Fq** | TATATCATCATCGTGGTGGGGATG | 181 bp | qPCR | **(23)** |
| **acrB-Rq** | TGTCTTTCTCTTTGGTCAGGAAGT |  | qPCR | **(23)** |
| **marB-Fq** | CTTGCTGGTGCTGGTTTCC | 100 bp | qPCR | **(23)** |
| **marB-Rq** | GTTTTGCGTGGACGGCATAA |  | qPCR | **(23)** |

**Table S1. Oligonucleotides used in this study.**

| **Number of strains** | **MIC values of TGC** | **1/2 MIC** | **Phenotype** | **FC *rob* expression** | **FC *acrA* expression** | **FC *acrB* expression** | **FC *acrB*/*acrA*** | **FC *ramA* expression** | **FC *soxS* expression** | **FC *marB* expression** | **FC *marA* gene expression** | **FC *tolC* gene expression** |
| --- | --- | --- | --- | --- | --- | --- | --- | --- | --- | --- | --- | --- |
| 1 | 0,25 | 0,125 | Susceptible | 0,52 | 2,12 | 4,6 | 2,17 | 32,6 | 0,56 | 0,0005 | 120,02 | 12,34 |
| 2 | 0,25 | 0,125 | Susceptible | 0,75 | 2,7 | 2,98 | 1,10 | 8,13 | 41,6 | 1,82 | 901,57 | 11,47 |
| 3 | 16 | 8 | Resistant | 2,42 | 1,99 | 24,53 | 12,33 | 7,09 | 73,28 | 5,37 | 1479,1 | 116,87 |
| 4 | 0,25 | 0,125 | Susceptible | 0,013 | 0,309 | 0,001 | 0,00 | 1,09 | 0,69 | 0,0002 | 44,157 | 9,1 |
| 5 | 0,25 | 0,125 | Susceptible | 0,49 | 1,27 | 0,01 | 0,01 | 16,58 | 7,76 | 0,07 | 218,78 | 2,1 |
| 6 | 8 | 4 | Resistant | 55,74 | 4,69 | 6,68 | 1,42 | 50,79 | 59,56 | 0,13 | 912,01 | 274,69 |
| 7 | 16 | 8 | Resistant | 2,72 | 8,07 | 10,34 | 1,28 | 36,32 | 7,94 | 6,16 | 4191 | 367,41 |
| 8 | 1 | 0,5 | Susceptible | 0,82 | 2,69 | 3,4 | 1,26 | 8,1 | 0,49 | 0,16 | 319,89 | 7,11 |
| 9 | 1 | 0,5 | Susceptible | 0,86 | 3,35 | 2,832 | 0,85 | 33,01 | 0,716 | 0,0082 | 426,58 | 8,45 |
| 10 | 1 | 0,5 | Susceptible | 1,21 | 1,6 | 2,49 | 1,56 | 31,25 | 1,41 | 0,04 | 226,46 | 3,36 |
| 11 | 8 | 4 | Resistant | 2,19 | 3,23 | 8,59 | 2,66 | 71,23 | 19,7 | 0,16 | 8912,4 | 874,29 |
| 12 | 0,25 | 0,125 | Susceptible | 0,6 | 2,23 | 1,03 | 0,46 | 11,71 | 4,37 | 0,2 | 1905,4 | 1,11 |
| 13 | 4 | 2 | Resistant | 2,01 | 4,94 | 3,37 | 0,68 | 81,99 | 48,98 | 0,1 | 5292,5 | 635,42 |
| 14 | 8 | 4 | Resistant | 2,29 | 3,09 | 1,56 | 0,50 | 65,35 | 7,67 | 280,1 | 5763 | 236,98 |
| 15 | 8 | 4 | Resistant | 2,01 | 2,62 | 5,05 | 1,93 | 57,9 | 7,41 | 83,4 | 4613,2 | 334,71 |
| 16 | 16 | 8 | Resistant | 2,101 | 15,51 | 14,331 | 0,92 | 141,23 | 1109 | 612,83 | 30000 | 321,67 |
| 17 | 8 | 4 | Resistant | 1,01 | 118,27 | 4,48 | 0,04 | 12,38 | 6,3 | 634,57 | 2822,7 | 784,85 |
| 18 | 32 | 16 | Resistant | 2,18 | 15,64 | 17,19 | 1,10 | 230,52 | 1216 | 1111,1 | 36728 | 744,12 |
| 19 | 0,25 | 0,125 | Susceptible | 0,74 | 8,42 | 0,56 | 0,07 | 31,704 | 288,4 | 8,61 | 5632 | 2,12 |
| 20 | 0,5 | 0,25 | Susceptible | 0,81 | 9,84 | 3,56 | 0,36 | 1,74 | 0,78 | 55,25 | 630,94 | 3,36 |
| 21 | 0,5 | 0,25 | Susceptible | 0,38 | 1,45 | 2,59 | 1,79 | 16,71 | 1,05 | 19,73 | 206,54 | 1,87 |
| 22 | 1 | 0,5 | Susceptible | 0,74 | 2,52 | 2,67 | 1,06 | 131,25 | 169,82 | 0,08 | 143,6 | 1,47 |
| 23 | 16 | 8 | Resistant | 1,61 | 20,69 | 5,08 | 0,25 | 10,17 | 213,8 | 1181 | 7224 | 369,87 |
| 24 | 8 | 4 | Resistant | 1,24 | 15,9 | 3,89 | 0,24 | 51,69 | 350,5 | 8656 | 2394,1 | 256,78 |
| 25 | 16 | 8 | Resistant | 6,73 | 55,14 | 70,88 | 1,29 | 898,97 | 3388 | 92,26 | 370000 | 222,3 |
| 26 | 0,5 | 0,25 | Susceptible | 0,05 | 1,87 | 1,84 | 0,98 | 51,36 | 218,78 | 0,05 | 1,29 | 3,36 |
| 28 | 0,5 | 0,25 | Susceptible | 0,52 | 0,27 | 1,4 | 5,19 | 0,04 | 1,6 | 0,03 | 1364,3 | 3,74 |
| 29 | 0,5 | 0,25 | Susceptible | 0,28 | 0,53 | 0,0005 | 0,00 | 0,06 | 0,65 | 1,26 | 1023,3 | 11,47 |
| 30 | 32 | 16 | Resistant | 2,79 | 47,69 | 22,59 | 0,47 | 89,46 | 2113 | 85,114 | 710000 | 1028,54 |
| 31 | 0,5 | 0,25 | Susceptible | 0,78 | 0,62 | 2,98 | 4,81 | 63,99 | 3,59 | 676,08 | 234,2 | 1,47 |
| 32 | 0,5 | 0,25 | Susceptible | 0,76 | 2,64 | 1,89 | 0,72 | 34,28 | 42,17 | 732,82 | 134,9 | 2,44 |
| 33 | 8 | 4 | Resistant | 2,33 | 32,34 | 2,65 | 0,08 | 61,07 | 245,47 | 9237,1 | 2654,5 | 898,6 |
| 34 | 4 | 2 | Resistant | 2,71 | 9,84 | 2,37 | 0,24 | 62,39 | 48,42 | 3981 | 5559 | 785,2 |
| 35 | 0,5 | 0,25 | Susceptible | 0,91 | 8,84 | 1,09 | 0,12 | 17,79 | 7,88 | 269,1 | 6,84 | 1,2 |
| 40 | 16 | 8 | Resistant | 1,79 | 18,84 | 3,73 | 0,20 | 16,87 | 16,63 | 805,47 | 477,36 | 854,1 |
| 44 | 8 | 4 | Resistant | 1,52 | 2,39 | 2,47 | 1,03 | 8,43 | 15,49 | 674,69 | 338,96 | 774,23 |
| 45 | 4 | 2 | Resistant | 1,51 | 7,54 | 3,6 | 0,48 | 22,64 | 10,94 | 393,67 | 1549 | 652,2 |
| 46 | 16 | 8 | Resistant | 1,74 | 10,9 | 4,15 | 0,38 | 23,42 | 194,98 | 2199,5 | 1414,7 | 693,87 |
| 47 | 8 | 4 | Resistant | 1,57 | 2,82 | 2,2 | 0,78 | 104,34 | 8,61 | 305,49 | 3499,3 | 1122,2 |
| 48 | 8 | 4 | Resistant | 1,02 | 3,64 | 1,24 | 0,34 | 92,89 | 12,74 | 8709,4 | 21878 | 1748,6 |
| 49 | 0,25 | 0,125 | Susceptible | 0,2 | 1,1 | 0,474 | 0,43 | 7,25 | 0,252 | 0,05 | 0,41 | 0,96 |
| 52 | 0,25 | 0,125 | Susceptible | 0,415 | 0,55 | 0,941 | 1,71 | 1,47 | 0,95 | 0,02 | 0,01 | 2,22 |
| 53 | 0,25 | 0,125 | Susceptible | 4,01 | 18,9 | 2,68 | 0,14 | 23,23 | 6,11 | 0,18 | 65,313 | 13,44 |
| 54 | 0,25 | 0,125 | Susceptible | 0,59 | 0,865 | 0,908 | 1,05 | 2,8 | 1,27 | 0,002 | 263,43 | 12,54 |
| 70 | 8 | 4 | Resistant | 1,13 | 4 | 1,52 | 0,38 | 60,99 | 0,69 | 120,23 | 1280,4 | 1985,6 |
| 71 | 8 | 4 | Resistant | 0,36 | 18,26 | 0,66 | 0,04 | 38,42 | 0,24 | 5069,4 | 3054,9 | 2365,3 |
| 72 | 8 | 4 | Resistant | 1,13 | 3,53 | 0,15 | 0,04 | 8,5 | 10,61 | 1396,4 | 467,74 | 2487,7 |
| 78 | 8 | 4 | Resistant | 0,37 | 3,46 | 3,53 | 1,02 | 8,44 | 1,18 | 3672,8 | 645,65 | 2134,7 |
| 79 | 8 | 4 | Resistant | 1,53 | 2,72 | 2,56 | 0,94 | 10,89 | 1,02 | 1333,5 | 204,17 | 2258,9 |
| 80 | 8 | 4 | Resistant | 0,49 | 2,62 | 2,64 | 1,01 | 15,71 | 0,98 | 398,11 | 223,87 | 287,4 |
| 81 | 8 | 4 | Resistant | 1,34 | 4,67 | 7,19 | 1,54 | 13,19 | 1,43 | 1333,5 | 363,08 | 2856,6 |
| 82 | 16 | 8 | Resistant | 0,2 | 5,55 | 7,36 | 1,33 | 4,47 | 0,19 | 7079,5 | 1335 | 287,5 |
| 87 | 16 | 8 | Resistant | 10,9 | 8,98 | 8,33 | 0,93 | 13,65 | 0,93 | 4168,7 | 218,78 | 336,98 |
| 99 | 16 | 8 | Resistant | 0,43 | 3,89 | 4,35 | 1,12 | 7,39 | 0,34 | 2985,4 | 257,04 | 256,89 |
| 100 | 32 | 16 | Resistant | 0,56 | 13,26 | 8,49 | 0,64 | 33,07 | 1,43 | 7852,4 | 1303,2 | 2368,7 |
| 101 | 16 | 8 | Resistant | 3,93 | 2,99 | 2,16 | 0,72 | 84,29 | 8,71 | 276,89 | 396,06 | 3347,4 |
| 102 | 8 | 4 | Resistant | 2,08 | 1,91 | 0,82 | 0,43 | 84,54 | 3,11 | 301,96 | 159,19 | 2874,1 |
| 103 | 32 | 16 | Resistant | 1,454 | 5,29 | 2,72 | 0,51 | 48,1 | 7,94 | 611,61 | 322,42 | 1124,5 |
| 104 | 8 | 4 | Resistant | 6,14 | 14,07 | 1,58 | 0,11 | 49,97 | 14 | 633,59 | 1061,8 | 1287,88 |
| 105 | 4 | 2 | Resistant | 1,1 | 4,39 | 0,79 | 0,18 | 23,24 | 4,04 | 385,99 | 238,34 | 1147,87 |

**Table S2. Fold-change (FC) expression values of all analyzed genes, tigecycline resistance phenotypes, and minimum inhibitory concentration (MIC) values for each clinical *Enterobacter cloacae* isolate under tigecycline exposure.**

| **Number of strains** | **MIC values of TGC** | **1/2 MIC** | **Phenotype** | **FC *rob* expression** | **FC *acrA* expression** | **FC *acrB* expression** | **FC *ramA* expression** | **FC *soxS* expression** | **FC *marB* expression** | **FC *marA* gene expression** | **FC *tolC* gene expression** |
| --- | --- | --- | --- | --- | --- | --- | --- | --- | --- | --- | --- |
| 1 | 0,25 | 0,125 | Susceptible | 2,36 | 2,15 | 4,08 | 3,261 | 23,71 | 2,48 | 3,12 | 0,977 |
| 2 | 0,25 | 0,125 | Susceptible | 1,37 | 2,01 | 2,74 | 3,41 | 13,71 | 2,18 | 3,33 | 0,74 |
| 3 | 16 | 8 | Resistant | 3,73 | 4,47 | 7,57 | 26,26 | 451,83 | 32,35 | 4,29 | 2,98 |
| 4 | 0,25 | 0,125 | Susceptible | 0,75 | 2,37 | 1,04 | 8,49 | 2,22 | 1,82 | 2,21 | 1,21 |
| 5 | 0,25 | 0,125 | Susceptible | 4,13 | 2,07 | 1,23 | 9,42 | 2,02 | 3,83 | 2,69 | 2,37 |
| 6 | 8 | 4 | Resistant | 3,72 | 2,74 | 7,86 | 3,43 | 334,96 | 12,2 | 4,44 | 20,18 |
| 7 | 16 | 8 | Resistant | 6,14 | 3,74 | 3,37 | 6,41 | 129,68 | 10,54 | 2,21 | 5,59 |
| 8 | 1 | 0,5 | Susceptible | 1,67 | 2,37 | 3,22 | 0,89 | 26,69 | 12,16 | 2,12 | 7,16 |
| 9 | 1 | 0,5 | Susceptible | 1,98 | 2,68 | 1,74 | 1,02 | 12,21 | 3,36 | 3,01 | 5,41 |
| 10 | 1 | 0,5 | Susceptible | 5,25 | 1,86 | 1,23 | 1,76 | 6,02 | 2,84 | 2,08 | 1,49 |
| 11 | 8 | 4 | Resistant | 3,62 | 3,05 | 10,75 | 26,08 | 358,9 | 27,22 | 3,33 | 6,69 |
| 12 | 0,25 | 0,125 | Susceptible | 1,73 | 2,36 | 1,53 | 7,52 | 1,38 | 9,77 | 1,25 | 2,01 |
| 13 | 4 | 2 | Resistant | 2,11 | 6,59 | 6,36 | 63,31 | 28,43 | 13,18 | 4,87 | 4,26 |
| 14 | 8 | 4 | Resistant | 3,12 | 4,58 | 2,23 | 8,54 | 7,71 | 6,52 | 5,15 | 6,52 |
| 15 | 8 | 4 | Resistant | 3,36 | 3,13 | 2,74 | 6,67 | 21,36 | 2,88 | 4,14 | 5,44 |
| 16 | 16 | 8 | Resistant | 3,03 | 4,09 | 6,19 | 6,56 | 40,73 | 3,46 | 3,36 | 10,06 |
| 17 | 8 | 4 | Resistant | 4,12 | 3,21 | 2,96 | 8,88 | 19,97 | 11,44 | 2,98 | 9,21 |
| 18 | 32 | 16 | Resistant | 3,9 | 3,45 | 3,01 | 42,6 | 15,62 | 13,69 | 3,69 | 14,45 |
| 19 | 0,25 | 0,125 | Susceptible | 4,69 | 2,02 | 2,54 | 6,3 | 12,64 | 3,31 | 2,1 | 3,01 |
| 20 | 0,5 | 0,25 | Susceptible | 5,55 | 1,63 | 1,99 | 1,17 | 6,21 | 4,14 | 2,5 | 3,46 |
| 21 | 0,5 | 0,25 | Susceptible | 1,45 | 2,41 | 1,07 | 3,66 | 10,12 | 6,32 | 3,06 | 5,52 |
| 22 | 1 | 0,5 | Susceptible | 2,14 | 3,01 | 1,03 | 3,54 | 8,2 | 4,1 | 4,12 | 1,01 |
| 23 | 16 | 8 | Resistant | 6,12 | 4,02 | 2,51 | 9,12 | 14,44 | 14,26 | 10,1 | 2,74 |
| 24 | 8 | 4 | Resistant | 3,47 | 4,4 | 2,23 | 10,48 | 16,23 | 11,2 | 2,09 | 6,26 |
| 25 | 16 | 8 | Resistant | 8,45 | 3,77 | 3,21 | 8,42 | 17,25 | 10,61 | 5,87 | 5,26 |
| 26 | 0,5 | 0,25 | Susceptible | 2,76 | 1,48 | 2,23 | 3,46 | 4,89 | 2,21 | 2,21 | 6,16 |
| 28 | 0,5 | 0,25 | Susceptible | 1,74 | 2,87 | 3,12 | 14,41 | 8,41 | 3,36 | 3,54 | 4,87 |
| 29 | 0,5 | 0,25 | Susceptible | 4,27 | 2,03 | 1,47 | 6,47 | 5,51 | 3,74 | 3,5 | 3,98 |
| 30 | 32 | 16 | Resistant | 1,62 | 2,26 | 2,81 | 9,44 | 24,26 | 20,41 | 4,1 | 5,33 |
| 31 | 0,5 | 0,25 | Susceptible | 1,23 | 1,06 | 2,71 | 1,22 | 3,85 | 1,74 | 7,1 | 1,45 |
| 32 | 0,5 | 0,25 | Susceptible | 1,87 | 1,51 | 2,01 | 1,24 | 2,01 | 2,67 | 5,93 | 2,6 |
| 33 | 8 | 4 | Resistant | 9,29 | 2,49 | 3,31 | 241,7 | 323,74 | 35,89 | 3,74 | 260,01 |
| 34 | 4 | 2 | Resistant | 6,61 | 3,21 | 2,31 | 4,3 | 19,63 | 36,51 | 6,38 | 31,2 |
| 35 | 0,5 | 0,25 | Susceptible | 1,25 | 1,13 | 2,04 | 1,21 | 6,21 | 2,54 | 2,65 | 3,1 |
| 40 | 16 | 8 | Resistant | 4,98 | 2,26 | 2,78 | 4,12 | 13,61 | 10,21 | 7,26 | 12,4 |
| 44 | 8 | 4 | Resistant | 37,39 | 2,86 | 4,05 | 58,73 | 72,44 | 16,98 | 7,41 | 26,1 |
| 45 | 4 | 2 | Resistant | 21,35 | 2,04 | 1,93 | 32,21 | 12,57 | 117,1 | 4,67 | 14,74 |
| 46 | 16 | 8 | Resistant | 10,08 | 2,87 | 5,91 | 45,51 | 22,64 | 34,27 | 2,98 | 33,84 |
| 47 | 8 | 4 | Resistant | 1,78 | 2,09 | 2,66 | 15,5 | 47,86 | 18,83 | 2,69 | 6,02 |
| 48 | 8 | 4 | Resistant | 7,41 | 2,74 | 2,12 | 22,47 | 12,3 | 41,52 | 5,41 | 4,93 |
| 49 | 0,25 | 0,125 | Susceptible | 2,04 | 1,56 | 2,41 | 3,45 | 4,48 | 12,63 | 2,07 | 3,36 |
| 52 | 0,25 | 0,125 | Susceptible | 0,53 | 0,78 | 1,84 | 10,61 | 3,58 | 1,75 | 2,13 | 2,44 |
| 53 | 0,25 | 0,125 | Susceptible | 3,72 | 2,36 | 2,17 | 4,14 | 14,2 | 3,17 | 1,76 | 10,1 |
| 54 | 0,25 | 0,125 | Susceptible | 1,26 | 2,47 | 2,09 | 1,7 | 9,22 | 11,27 | 1,01 | 12,24 |
| 70 | 8 | 4 | Resistant | 3,07 | 3,69 | 6,22 | 6,5 | 124,45 | 46,77 | 6,38 | 6,38 |
| 71 | 8 | 4 | Resistant | 1,24 | 2,07 | 2,45 | 12,18 | 27,22 | 2,04 | 3,35 | 3,19 |
| 72 | 8 | 4 | Resistant | 5,4 | 3,13 | 2,36 | 11,45 | 124,01 | 37,12 | 4,57 | 3,25 |
| 78 | 8 | 4 | Resistant | 4,1 | 2,1 | 2,78 | 10,23 | 63,67 | 12,67 | 2,36 | 7,41 |
| 79 | 8 | 4 | Resistant | 3,07 | 3,42 | 6,8 | 51,99 | 67,08 | 53,08 | 2,89 | 12,64 |
| 80 | 8 | 4 | Resistant | 2,3 | 2,24 | 2,16 | 3,54 | 54,87 | 25,3 | 2,33 | 13,37 |
| 81 | 8 | 4 | Resistant | 3,01 | 1,95 | 1,87 | 2,09 | 39,51 | 24,71 | 6,54 | 19,65 |
| 82 | 16 | 8 | Resistant | 5,08 | 1,78 | 1,92 | 2,56 | 28,97 | 16,34 | 2,46 | 14,7 |
| 87 | 16 | 8 | Resistant | 4,16 | 2,08 | 2,19 | 2,88 | 34,46 | 8,96 | 3,91 | 6,78 |
| 99 | 16 | 8 | Resistant | 2,28 | 2,12 | 1,93 | 2,45 | 23,54 | 7,72 | 2,46 | 6,32 |
| 100 | 32 | 16 | Resistant | 6,12 | 2,97 | 2,32 | 12,65 | 74,89 | 31,25 | 2,87 | 8,02 |
| 101 | 16 | 8 | Resistant | 2,54 | 1,87 | 1,74 | 3,14 | 21,74 | 15,41 | 3,52 | 15,32 |
| 102 | 8 | 4 | Resistant | 5,64 | 3,12 | 2,58 | 12,69 | 162,27 | 41,02 | 8,91 | 21,1 |
| 103 | 32 | 16 | Resistant | 3,32 | 2,54 | 1,27 | 4,36 | 6,82 | 5,68 | 3,69 | 8,19 |
| 104 | 8 | 4 | Resistant | 2,87 | 2,22 | 2,22 | 5,62 | 22,12 | 12,79 | 4,12 | 15,41 |
| 105 | 4 | 2 | Resistant | 4,2 | 2,96 | 3,04 | 11,63 | 39,61 | 12,47 | 4,4 | 9,54 |

**Table S3. Fold-change (FC) expression values of all analyzed genes, tigecycline resistance phenotypes, and minimum inhibitory concentration (MIC) values for each clinical *Enterobacter cloacae* isolate under standard conditions.**

| **Number of strains** | **log_2_FC *rob* expression** | **log_2_FC *acrA* expression** | **log_2_FC *acrB* expression** | **log_2_FC *acrB*/*acrA*** | **log_2_FC *ramA* expression** | **log_2_FC *soxS* expression** | **log_2_FC *marB* expression** | **log_2_FC *marA* gene expression** | **log_2_FC *tolC* gene expression** |
| --- | --- | --- | --- | --- | --- | --- | --- | --- | --- |
| 3 | 1,27501 | 0,99277 | 4,61648 | 3,62371 | 2,82579 | 6,19535 | 2,42492 | 10,5305 | 6,868760834 |
| 6 | 5,80064 | 2,22959 | 2,73985 | 0,51026 | 5,66647 | 5,89627 | -2,94342 | 9,83291 | 8,10166058 |
| 7 | 1,44361 | 3,01257 | 3,37016 | 0,3576 | 5,18269 | 2,98914 | 2,62293 | 12,0331 | 8,521247083 |
| 11 | 1,13093 | 1,69153 | 3,10266 | 1,41112 | 6,15441 | 4,30012 | -2,64386 | 13,1216 | 9,771968088 |
| 13 | 1,0072 | 2,30451 | 1,75275 | -0,55176 | 6,35738 | 5,61412 | -3,32193 | 12,3697 | 9,31156669 |
| 14 | 1,19535 | 1,62761 | 0,64155 | -0,98606 | 6,03012 | 2,93923 | 8,1298 | 12,4926 | 7,888621497 |
| 15 | 1,0072 | 1,38957 | 2,33628 | 0,94672 | 5,85549 | 2,88947 | 6,38198 | 12,1716 | 8,386767844 |
| 16 | 1,07108 | 3,95513 | 3,84107 | -0,11406 | 7,1419 | 10,115 | 9,25934 | 14,8727 | 8,329437582 |
| 17 | 0,01436 | 6,88594 | 2,1635 | -4,72244 | 3,62994 | 2,65535 | 9,30964 | 11,4629 | 9,616273143 |
| 18 | 1,12433 | 3,96717 | 4,1035 | 0,13633 | 7,84875 | 10,2479 | 10,1178 | 15,1646 | 9,539391485 |
| 23 | 0,68706 | 4,37086 | 2,34483 | -2,02603 | 3,34625 | 7,74012 | 10,2058 | 12,8186 | 8,530874479 |
| 24 | 0,31034 | 3,99095 | 1,95977 | -2,03118 | 5,69181 | 8,45327 | 13,0795 | 11,2253 | 8,004389028 |
| 25 | 2,75061 | 5,78503 | 6,14731 | 0,36228 | 9,81213 | 11,7262 | 6,52763 | 18,4972 | 7,796364138 |
| 30 | 1,48027 | 5,57561 | 4,49761 | -1,078 | 6,48317 | 11,0451 | 6,41132 | 19,4375 | 10,00638219 |
| 33 | 1,22033 | 5,01525 | 1,40599 | -3,60926 | 5,93239 | 7,9394 | 13,1732 | 11,3742 | 9,811535252 |
| 34 | 1,43829 | 3,29866 | 1,24489 | -2,05377 | 5,96324 | 5,59753 | 11,9589 | 12,4406 | 9,616916363 |
| 40 | 0,83996 | 4,23573 | 1,89918 | -2,33655 | 4,07639 | 4,05572 | 9,65369 | 8,89893 | 9,738261184 |
| 44 | 0,60407 | 1,25701 | 1,30451 | 0,0475 | 3,07553 | 3,95327 | 9,39808 | 8,40497 | 9,5966184 |
| 45 | 0,59455 | 2,91456 | 1,848 | -1,06657 | 4,5008 | 3,45154 | 8,62084 | 10,5971 | 9,349170631 |
| 46 | 0,79909 | 3,44626 | 2,05311 | -1,39314 | 4,54967 | 7,60718 | 11,103 | 10,4663 | 9,438521582 |
| 47 | 0,65076 | 1,4957 | 1,1375 | -0,35819 | 6,70515 | 3,10601 | 8,25498 | 11,7729 | 10,1321141 |
| 48 | 0,02857 | 1,86394 | 0,31034 | -1,5536 | 6,53745 | 3,67129 | 13,0884 | 14,4172 | 10,77198459 |
| 70 | 0,17632 | 2 | 0,60407 | -1,39593 | 5,9305 | -0,53533 | 6,90965 | 10,3224 | 10,95535931 |
| 71 | -1,47393 | 4,19061 | -0,59946 | -4,79008 | 5,26379 | -2,05889 | 12,3076 | 11,5769 | 11,20780746 |
| 72 | 0,17632 | 1,81967 | -2,73697 | -4,55663 | 3,08746 | 3,40735 | 10,4475 | 8,86956 | 11,2805968 |
| 78 | -1,4344 | 1,79077 | 1,81967 | 0,0289 | 3,07724 | 0,23879 | 11,8427 | 9,33461 | 11,05981762 |
| 79 | 0,61353 | 1,44361 | 1,35614 | -0,08746 | 3,44493 | 0,02857 | 10,381 | 7,67363 | 11,14140469 |
| 80 | -1,02915 | 1,38957 | 1,40054 | 0,01097 | 3,97361 | -0,02915 | 8,63702 | 7,80652 | 8,166916252 |
| 81 | 0,42223 | 2,22342 | 2,84599 | 0,62257 | 3,72137 | 0,51602 | 10,381 | 8,50414 | 11,48008332 |
| 82 | -2,32193 | 2,47249 | 2,87971 | 0,40722 | 2,16027 | -2,39593 | 12,7894 | 10,3826 | 8,167418146 |
| 87 | 3,44626 | 3,16672 | 3,05832 | -0,1084 | 3,77083 | -0,1047 | 12,0254 | 7,77334 | 8,396519159 |
| 99 | -1,21759 | 1,95977 | 2,12102 | 0,16125 | 2,88557 | -1,55639 | 11,5437 | 8,00585 | 8,005006921 |
| 100 | -0,8365 | 3,72901 | 3,08576 | -0,64324 | 5,04745 | 0,51602 | 12,9389 | 10,3478 | 11,20987977 |
| 101 | 1,97453 | 1,58015 | 1,11103 | -0,46911 | 6,39729 | 3,12267 | 8,11317 | 8,62958 | 11,70882524 |
| 102 | 1,05658 | 0,93357 | -0,2863 | -1,21988 | 6,40156 | 1,63691 | 8,23821 | 7,31461 | 11,48889454 |
| 103 | 0,54003 | 2,40327 | 1,44361 | -0,95966 | 5,58796 | 2,98914 | 9,25647 | 8,3328 | 10,13506795 |
| 104 | 2,61824 | 3,81455 | 0,65992 | -3,15463 | 5,64299 | 3,80735 | 9,30741 | 10,0523 | 10,33078246 |
| 105 | 0,1375 | 2,13422 | -0,34008 | -2,4743 | 4,53854 | 2,01436 | 8,59242 | 7,89688 | 10,16474355 |

**Table S4. Log_2_ fold change (log_2_FC) values of gene expression in tigecycline-resistant *Enterobacter cloacae* isolates under tigecycline exposure. The table includes efflux pump, and regulatory genes analyzed in this study.**

| **Number of strains** | **log_2_FC *rob* expression** | **log_2_FC *acrA* expression** | **log_2_FC *acrB* expression** | **log_2_FC *acrB*/*acrA*** | **log_2_FC *ramA* expression** | **log_2_FC *soxS* expression** | **log_2_FC *marB* expression** | **log_2_FC *marA* gene expression** | **log_2_FC *tolC* gene expression** |
| --- | --- | --- | --- | --- | --- | --- | --- | --- | --- |
| 1 | -0,94342 | 1,08406 | 2,20163 | 1,11757 | 5,0268 | -0,8365 | -10,9658 | 6,90713 | 3,625270489 |
| 2 | -0,41504 | 1,43296 | 1,57531 | 0,14235 | 3,02326 | 5,37851 | 0,86394 | 9,8163 | 3,519793486 |
| 4 | -6,26534 | -1,69432 | -9,96578 | -8,27146 | 0,12433 | -0,53533 | -12,2877 | 5,46457 | 3,185866545 |
| 5 | -1,02915 | 0,34483 | -6,64386 | -6,98868 | 4,05137 | 2,95606 | -3,8365 | 7,77334 | 1,070389328 |
| 8 | -0,2863 | 1,42761 | 1,76553 | 0,33793 | 3,01792 | -1,02915 | -2,64386 | 8,32143 | 2,82984956 |
| 9 | -0,21759 | 1,74416 | 1,50182 | -0,24234 | 5,04483 | -0,48197 | -6,93016 | 8,73667 | 3,078951341 |
| 10 | 0,27501 | 0,67807 | 1,31615 | 0,63807 | 4,96578 | 0,4957 | -4,64386 | 7,82311 | 1,748461233 |
| 12 | -0,73697 | 1,15704 | 0,04264 | -1,1144 | 3,54967 | 2,12763 | -2,32193 | 10,8959 | 0,150559677 |
| 19 | -0,4344 | 3,07382 | -0,8365 | -3,91032 | 4,98659 | 8,17193 | 3,10601 | 12,4594 | 1,084064265 |
| 20 | -0,30401 | 3,29866 | 1,83188 | -1,46678 | 0,79909 | -0,35845 | 5,7879 | 9,30136 | 1,748461233 |
| 21 | -1,39593 | 0,53605 | 1,37295 | 0,8369 | 4,06264 | 0,07039 | 4,30232 | 7,69028 | 0,90303827 |
| 22 | -0,4344 | 1,33342 | 1,41684 | 0,08342 | 7,03617 | 7,40786 | -3,64386 | 7,16591 | 0,555816155 |
| 26 | -4,32193 | 0,90304 | 0,87971 | -0,02333 | 5,68257 | 7,77334 | -4,32193 | 0,36737 | 1,748461233 |
| 28 | -0,94342 | -1,88897 | 0,48543 | 2,3744 | -4,64386 | 0,67807 | -5,05889 | 10,4139 | 1,90303827 |
| 29 | -1,8365 | -0,91594 | -10,9658 | -10,0498 | -4,05889 | -0,62149 | 0,33342 | 9,99901 | 3,519793486 |
| 31 | -0,35845 | -0,68966 | 1,57531 | 2,26497 | 5,99977 | 1,84398 | 9,40105 | 7,8716 | 0,555816155 |
| 32 | -0,39593 | 1,40054 | 0,91839 | -0,48215 | 5,0993 | 5,39815 | 9,51732 | 7,07575 | 1,286881148 |
| 35 | -0,13606 | 3,14405 | 0,12433 | -3,01972 | 4,15299 | 2,9782 | 8,072 | 2,774 | 0,263034406 |
| 49 | -2,32193 | 0,1375 | -1,07704 | -1,21454 | 2,85798 | -1,9885 | -4,32193 | -1,2863 | -0,058893689 |
| 52 | -1,26882 | -0,8625 | -0,08773 | 0,77476 | 0,55582 | -0,074 | -5,64386 | -6,64386 | 1,150559677 |
| 53 | 2,0036 | 4,24031 | 1,42223 | -2,81808 | 4,53792 | 2,61117 | -2,47393 | 6,0293 | 3,748461233 |
| 54 | -0,76121 | -0,20923 | -0,13924 | 0,06999 | 1,48543 | 0,34483 | -8,96578 | 8,04128 | 3,648465443 |

**Table S5. Log_2_ fold change (log_2_FC) values of gene expression in tigecycline-susceptible *Enterobacter cloacae* isolates under tigecycline exposure. The table includes efflux pump, and regulatory genes analyzed in this study.**

| **Number of strains** | **log_2_FC *rob* expression** | **log_2_FC *acrA* expression** | **log_2_FC *acrB* expression** | **log_2_FC *acrB*/*acrA*** | **log_2_FC *ramA* expression** | **log_2_FC *soxS* expression** | **log_2_FC *marB* expression** | **log_2_FC *marA* gene expression** | **log_2_FC *tolC* gene expression** |
| --- | --- | --- | --- | --- | --- | --- | --- | --- | --- |
| **3** | 1,90 | 2,16 | 2,92 | 0,76 | 4,71 | 8,82 | 5,02 | 2,10 | 1,58 |
| **6** | 1,90 | 1,45 | 2,97 | 1,52 | 1,78 | 8,39 | 3,61 | 2,15 | 4,33 |
| **7** | 2,62 | 1,90 | 1,75 | -0,15 | 2,68 | 7,02 | 3,40 | 1,14 | 2,48 |
| **11** | 1,86 | 1,61 | 3,43 | 1,82 | 4,70 | 8,49 | 4,77 | 1,74 | 2,74 |
| **13** | 1,08 | 2,72 | 2,67 | -0,05 | 5,98 | 4,83 | 3,72 | 2,28 | 2,09 |
| **14** | 1,64 | 2,20 | 1,16 | -1,04 | 3,09 | 2,95 | 2,70 | 2,36 | 2,70 |
| **15** | 1,75 | 1,65 | 1,45 | -0,19 | 2,74 | 4,42 | 1,53 | 2,05 | 2,44 |
| **16** | 1,60 | 2,03 | 2,63 | 0,60 | 2,71 | 5,35 | 1,79 | 1,75 | 3,33 |
| **17** | 2,04 | 1,68 | 1,57 | -0,12 | 3,15 | 4,32 | 3,52 | 1,58 | 3,20 |
| **18** | 1,96 | 1,79 | 1,59 | -0,20 | 5,41 | 3,97 | 3,78 | 1,88 | 3,85 |
| **23** | 2,61 | 2,01 | 1,33 | -0,68 | 3,19 | 3,85 | 3,83 | 3,34 | 1,45 |
| **24** | 1,79 | 2,14 | 1,16 | -0,98 | 3,39 | 4,02 | 3,49 | 1,06 | 2,65 |
| **25** | 3,08 | 1,91 | 1,68 | -0,23 | 3,07 | 4,11 | 3,41 | 2,55 | 2,40 |
| **30** | 0,70 | 1,18 | 1,49 | 0,31 | 3,24 | 4,60 | 4,35 | 2,04 | 2,41 |
| **33** | 3,22 | 1,32 | 1,73 | 0,41 | 7,92 | 8,34 | 5,17 | 1,90 | 8,02 |
| **34** | 2,72 | 1,68 | 1,21 | -0,47 | 2,10 | 4,29 | 5,19 | 2,67 | 4,96 |
| **40** | 2,32 | 1,18 | 1,48 | 0,30 | 2,04 | 3,77 | 3,35 | 2,86 | 3,63 |
| **44** | 5,22 | 1,52 | 2,02 | 0,50 | 5,88 | 6,18 | 4,09 | 2,89 | 4,71 |
| **45** | 4,42 | 1,03 | 0,95 | -0,08 | 5,01 | 3,65 | 6,87 | 2,22 | 3,88 |
| **46** | 3,33 | 1,52 | 2,56 | 1,04 | 5,51 | 4,50 | 5,10 | 1,58 | 5,08 |
| **47** | 0,83 | 1,06 | 1,41 | 0,35 | 3,95 | 5,58 | 4,23 | 1,43 | 2,59 |
| **48** | 2,89 | 1,45 | 1,08 | -0,37 | 4,49 | 3,62 | 5,38 | 2,44 | 2,30 |
| **70** | 1,62 | 1,88 | 2,64 | 0,75 | 2,70 | 6,96 | 5,55 | 2,67 | 2,67 |
| **71** | 0,31 | 1,05 | 1,29 | 0,24 | 3,61 | 4,77 | 1,03 | 1,74 | 1,67 |
| **72** | 2,43 | 1,65 | 1,24 | -0,41 | 3,52 | 6,95 | 5,21 | 2,19 | 1,70 |
| **78** | 2,04 | 1,07 | 1,48 | 0,40 | 3,35 | 5,99 | 3,66 | 1,24 | 2,89 |
| **79** | 1,62 | 1,77 | 2,77 | 0,99 | 5,70 | 6,07 | 5,73 | 1,53 | 3,66 |
| **80** | 1,20 | 1,16 | 1,11 | -0,05 | 1,82 | 5,78 | 4,66 | 1,22 | 3,74 |
| **81** | 1,59 | 0,96 | 0,90 | -0,06 | 1,06 | 5,30 | 4,63 | 2,71 | 4,30 |
| **82** | 2,34 | 0,83 | 0,94 | 0,11 | 1,36 | 4,86 | 4,03 | 1,30 | 3,88 |
| **87** | 2,06 | 1,06 | 1,13 | 0,07 | 1,53 | 5,11 | 3,16 | 1,97 | 2,76 |
| **99** | 1,19 | 1,08 | 0,95 | -0,14 | 1,29 | 4,56 | 2,95 | 1,30 | 2,66 |
| **100** | 2,61 | 1,57 | 1,21 | -0,36 | 3,66 | 6,23 | 4,97 | 1,52 | 3,00 |
| **101** | 1,34 | 0,90 | 0,80 | -0,10 | 1,65 | 4,44 | 3,95 | 1,82 | 3,94 |
| **102** | 2,50 | 1,64 | 1,37 | -0,27 | 3,67 | 7,34 | 5,36 | 3,16 | 4,40 |
| **103** | 1,73 | 1,34 | 0,34 | -1,00 | 2,12 | 2,77 | 2,51 | 1,88 | 3,03 |
| **104** | 1,52 | 1,15 | 1,15 | 0,00 | 2,49 | 4,47 | 3,68 | 2,04 | 3,95 |
| **105** | 2,07 | 1,57 | 1,60 | 0,04 | 3,54 | 5,31 | 3,64 | 2,14 | 3,25 |

**Table S6. Log_2_ fold change (log_2_FC) values of gene expression in tigecycline-resistant *Enterobacter cloacae* isolates under standard conditions. The table includes efflux pump, and regulatory genes analyzed in this study.**

| **Number of strains** | **log_2_FC *rob* expression** | **log_2_FC *acrA* expression** | **log_2_FC *acrB* expression** | **log_2_FC *acrB*/*acrA*** | **log_2_FC *ramA* expression** | **log_2_FC *soxS* expression** | **log_2_FC *marB* expression** | **log_2_FC *marA* gene expression** | **log_2_FC *tolC* gene expression** |
| --- | --- | --- | --- | --- | --- | --- | --- | --- | --- |
| **1** | 1,24 | 1,10 | 2,03 | 0,92 | 1,71 | 4,57 | 1,31 | 1,64 | -0,03 |
| **2** | 0,45 | 1,01 | 1,45 | 0,45 | 1,77 | 3,78 | 1,12 | 1,74 | -0,43 |
| **4** | -0,42 | 1,24 | 0,06 | -1,19 | 3,09 | 1,15 | 0,86 | 1,14 | 0,28 |
| **5** | 2,05 | 1,05 | 0,30 | -0,75 | 3,24 | 1,01 | 1,94 | 1,43 | 1,24 |
| **8** | 0,74 | 1,24 | 1,69 | 0,44 | -0,17 | 4,74 | 3,60 | 1,08 | 2,84 |
| **9** | 0,99 | 1,42 | 0,80 | -0,62 | 0,03 | 3,61 | 1,75 | 1,59 | 2,44 |
| **10** | 2,39 | 0,90 | 0,30 | -0,60 | 0,82 | 2,59 | 1,51 | 1,06 | 0,58 |
| **12** | 0,79 | 1,24 | 0,61 | -0,63 | 2,91 | 0,46 | 3,29 | 0,32 | 1,01 |
| **19** | 2,23 | 1,01 | 1,34 | 0,33 | 2,66 | 3,66 | 1,73 | 1,07 | 1,59 |
| **20** | 2,47 | 0,70 | 0,99 | 0,29 | 0,23 | 2,63 | 2,05 | 1,32 | 1,79 |
| **21** | 0,54 | 1,27 | 0,10 | -1,17 | 1,87 | 3,34 | 2,66 | 1,61 | 2,46 |
| **22** | 1,10 | 1,59 | 0,04 | -1,55 | 1,82 | 3,04 | 2,04 | 2,04 | 0,01 |
| **26** | 1,46 | 0,57 | 1,16 | 0,59 | 1,79 | 2,29 | 1,14 | 1,14 | 2,62 |
| **28** | 0,80 | 1,52 | 1,64 | 0,12 | 3,85 | 3,07 | 1,75 | 1,82 | 2,28 |
| **29** | 2,09 | 1,02 | 0,56 | -0,47 | 2,69 | 2,46 | 1,90 | 1,81 | 1,99 |
| **31** | 0,30 | 0,08 | 1,44 | 1,35 | 0,29 | 1,94 | 0,80 | 2,83 | 0,54 |
| **32** | 0,90 | 0,59 | 1,01 | 0,41 | 0,31 | 1,01 | 1,42 | 2,57 | 1,38 |
| **35** | 0,32 | 0,18 | 1,03 | 0,85 | 0,28 | 2,63 | 1,34 | 1,41 | 1,63 |
| **49** | 1,03 | 0,64 | 1,27 | 0,63 | 1,79 | 2,16 | 3,66 | 1,05 | 1,75 |
| **52** | -0,92 | -0,36 | 0,88 | 1,24 | 3,41 | 1,84 | 0,81 | 1,09 | 1,29 |
| **53** | 1,90 | 1,24 | 1,12 | -0,12 | 2,05 | 3,83 | 1,66 | 0,82 | 3,34 |
| **54** | 0,33 | 1,30 | 1,06 | -0,24 | 0,77 | 3,20 | 3,49 | 0,01 | 3,61 |

**Table S7. Log_2_ fold change (log_2_FC) values of gene expression in tigecycline-susceptible *Enterobacter cloacae* isolates under tigecycline exposure. The table includes efflux pump, and regulatory genes analyzed in this study.**


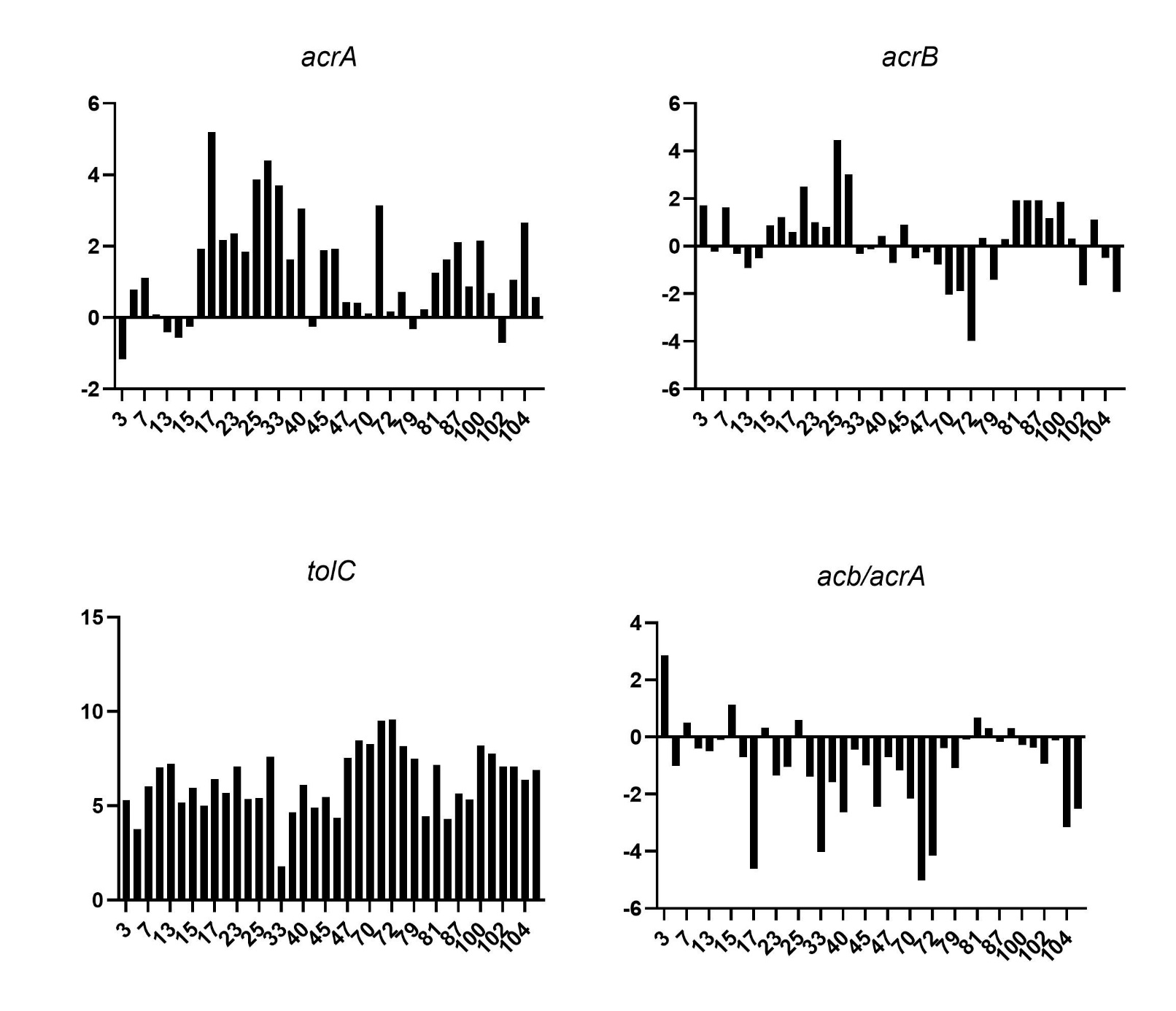


**Figure S1. Delta distance values illustrating the overall transcriptional divergence between tigecycline-exposed and standard conditions in resistant *Enterobacter cloacae* isolates. Each bar represents the calculated distance between paired samples, highlighting the magnitude of gene expression changes induced by tigecycline exposure.**


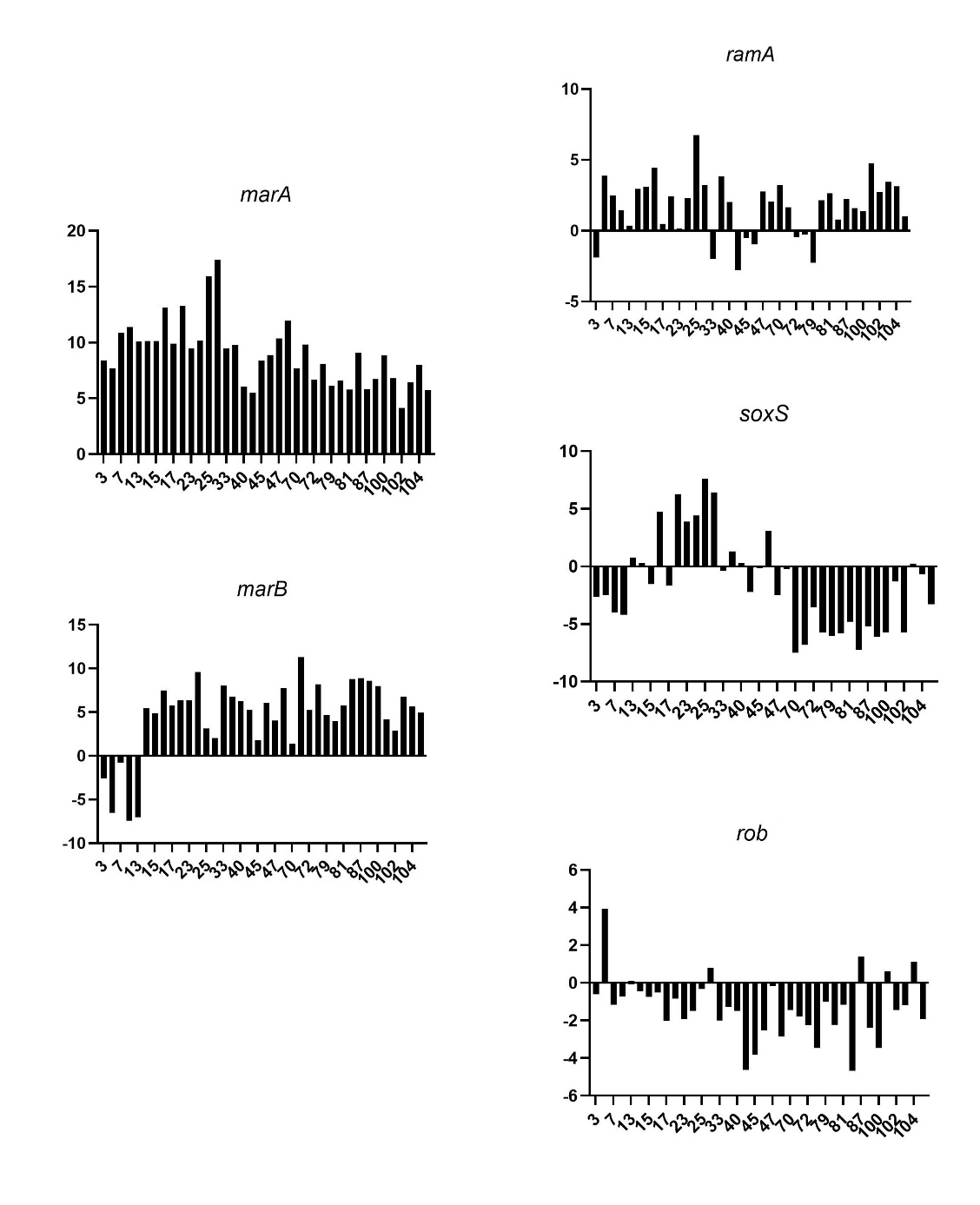


**Figure S2. Delta distance values illustrating the overall transcriptional divergence of regulatory genes between tigecycline-exposed and standard conditions in resistant *Enterobacter cloacae* isolates. Each bar represents the calculated distance between paired samples, highlighting the magnitude of gene expression changes induced by tigecycline exposure.**


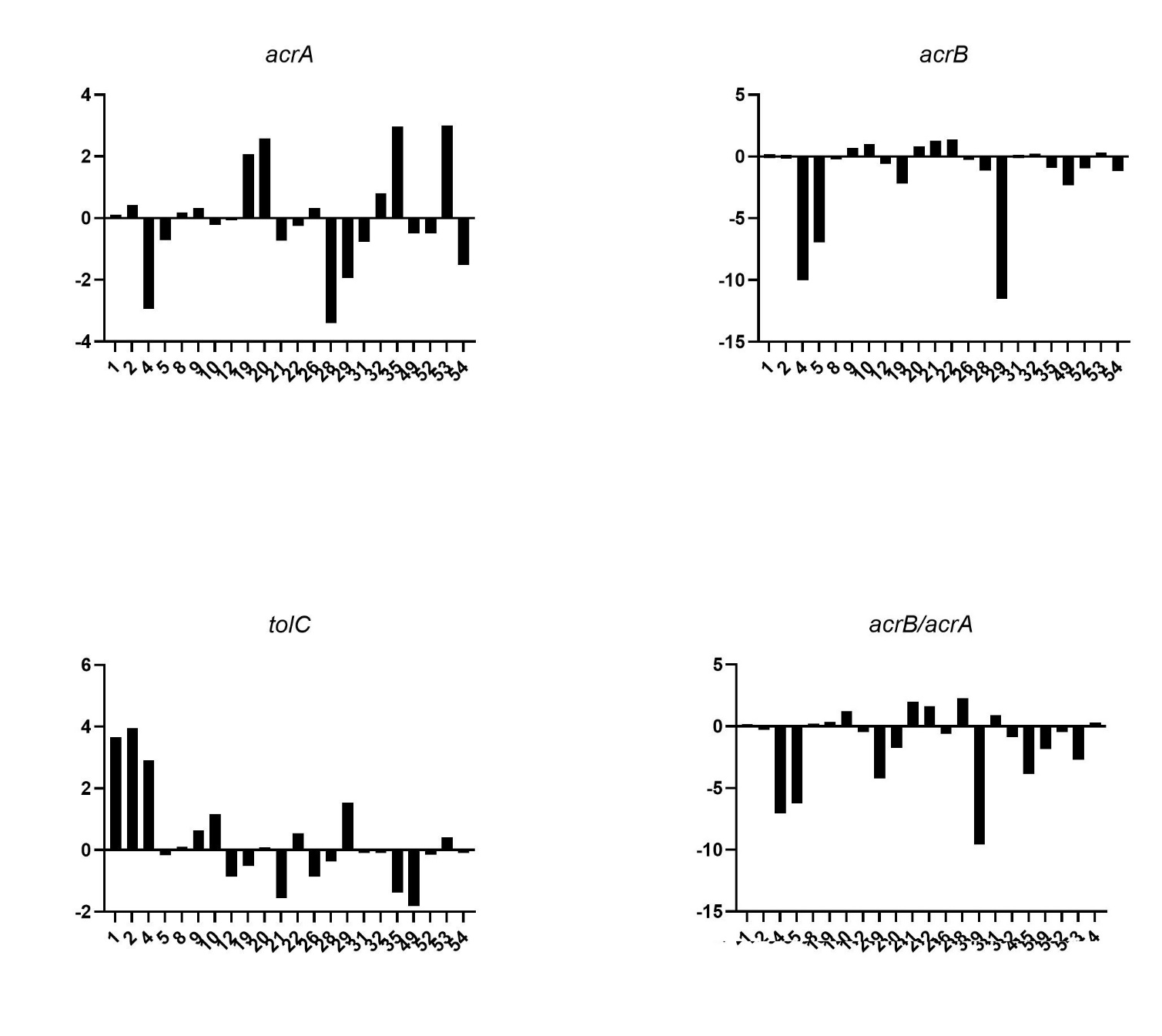


**Figure S3. Delta distance values illustrating the overall transcriptional divergence of efflux genes between tigecycline-exposed and standard conditions in susceptible *Enterobacter cloacae* isolates. Each bar represents the calculated distance between paired samples, highlighting the magnitude of gene expression changes in efflux gene systems induced by tigecycline exposure.**


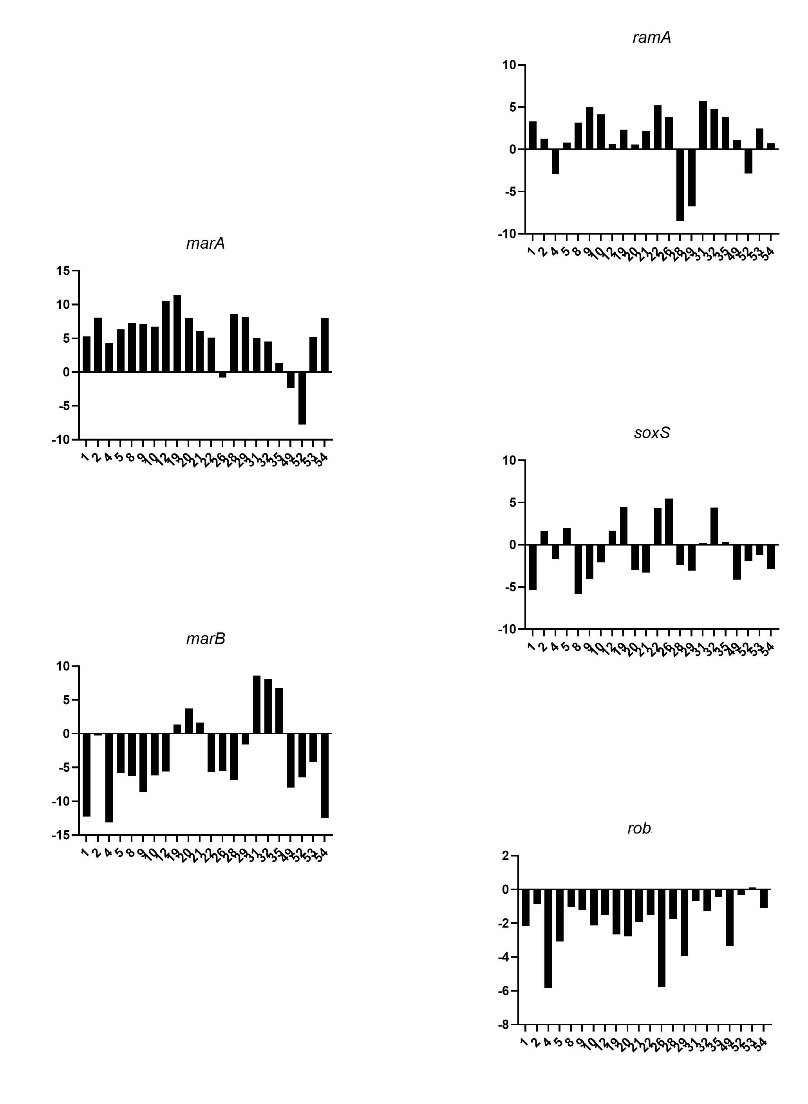


**Figure S4. Delta distance values illustrating the overall transcriptional divergence of regulatory genes between tigecycline-exposed and standard conditions in susceptible *Enterobacter cloacae* isolates. Each point represents the calculated distance between paired samples, highlighting the magnitude of gene expression changes in key regulatory pathways induced by tigecycline exposure.**
